# SFyNCS detects oncogenic fusions involving non-coding sequences in cancer

**DOI:** 10.1093/nar/gkad705

**Published:** 2023-08-28

**Authors:** Xiaoming Zhong, Jingyun Luan, Anqi Yu, Anna Lee-Hassett, Yuxuan Miao, Lixing Yang

**Affiliations:** Ben May Department for Cancer Research, University of Chicago, Chicago, IL, USA; Ben May Department for Cancer Research, University of Chicago, Chicago, IL, USA; Ben May Department for Cancer Research, University of Chicago, Chicago, IL, USA; Ben May Department for Cancer Research, University of Chicago, Chicago, IL, USA; Ben May Department for Cancer Research, University of Chicago, Chicago, IL, USA; University of Chicago Comprehensive Cancer Center, Chicago, IL, USA; Ben May Department for Cancer Research, University of Chicago, Chicago, IL, USA; University of Chicago Comprehensive Cancer Center, Chicago, IL, USA; Department of Human Genetics, University of Chicago, Chicago IL, USA

## Abstract

Fusion genes are well-known cancer drivers. However, most known oncogenic fusions are protein-coding, and very few involve non-coding sequences due to lack of suitable detection tools. We develop SFyNCS to detect fusions of both protein-coding genes and non-coding sequences from transcriptomic sequencing data. The main advantage of this study is that we use somatic structural variations detected from genomic data to validate fusions detected from transcriptomic data. This allows us to comprehensively evaluate various fusion detection and filtering strategies and parameters. We show that SFyNCS has superior sensitivity and specificity over existing algorithms through extensive benchmarking in cancer cell lines and patient samples. We then apply SFyNCS to 9565 tumor samples across 33 tumor types in The Cancer Genome Atlas cohort and detect a total of 165,139 fusions. Among them, 72% of the fusions involve non-coding sequences. We find a long non-coding RNA to recurrently fuse with various oncogenes in 3% of prostate cancers. In addition, we discover fusions involving two non-coding RNAs in 32% of dedifferentiated liposarcomas and experimentally validated the oncogenic functions in mouse model.

## Graphical Abstract

**Graphical Abstract:**
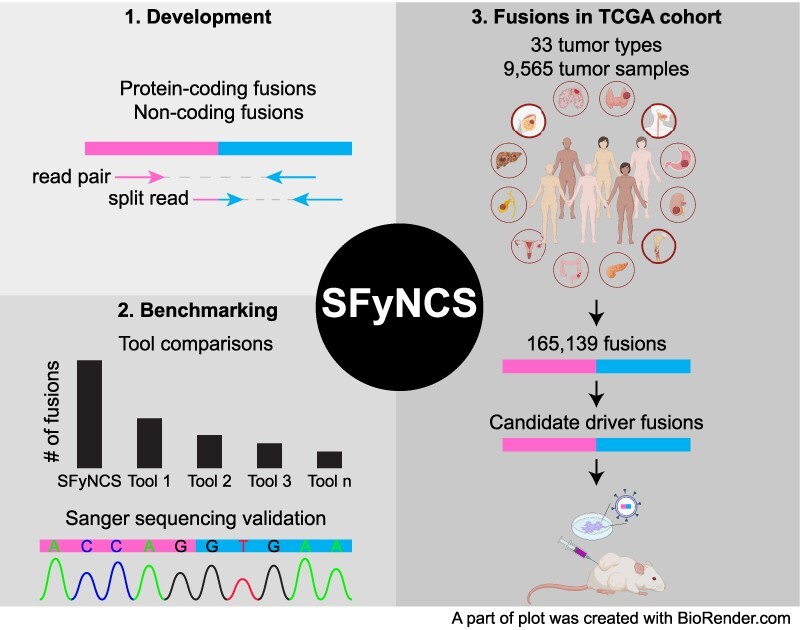

## INTRODUCTION

Fusions between protein-coding genes caused by somatic structural variations (SVs) are well-known cancer drivers (1,2), including *BCR*-*ABL1*, *EWS*-*FLI1*, *PML*-*RARA*, *TMPRSS2*-*ERG* and *FGFR3*-*TACC3*. It is estimated that 16% of cancers are driven by fusions (3). Fusion proteins represent ideal drug targets since tumor cell proliferation depends on them, but they do not exist in normal cells. One of the first targeted-therapy drugs in cancer, imatinib (Gleevec), is a small molecule inhibitor targeting the *BCR-ABL1* fusion protein (4). Many other inhibitors targeting different fusion proteins have since been approved for clinical use (5). To date, more than 1000 cancer-driving protein-coding fusions have been discovered (6). However, only several oncogenic non-coding fusions have been reported, including *HERV-K*-*ETV1* (7), *GAS5*-*BCL6* (8), *USP9Y*-*TTTY15* (9), *MALAT1*-*GLI1* (10), *TTYH1*-*C19MC* (11), *KDM4B*-*G039927* and *EPS15L1*-*lncOR7C2-1* (12). A previous study on over 9000 tumors from The Cancer Genome Atlas (TCGA) reported that only 4% of fusions involve non-coding sequences (3). This is because the algorithm used in that study, STAR-Fusion (3), was designed to mainly detect protein-coding fusions; therefore, the proportion of fusions involving non-coding sequences being 4% was certainly an underestimation. Fusions involving non-coding sequences are of clinical significance, as they can be used as biomarkers (13), and studies are ongoing to target them therapeutically (14,15). The discovery and characterization of non-coding fusions may reveal new disease mechanisms and novel drug targets.

It is extremely challenging to differentiate true fusions from artifacts. Chimeric molecules in the sequencing library, sequencing errors, alignment errors and read-through fusions further complicate fusion detection. Most existing fusion callers depend on annotations of protein-coding genes and non-coding RNAs (ncRNAs), including DEEPEST (16) and Arriba (17). However, current ncRNA databases are still far from ideal because many ncRNAs are expressed at low levels and are highly tissue specific. The low expression also poses a major challenge to detect fusions involving non-coding sequences. Therefore, known oncogenic non-coding fusions remain rare. Another major roadblock is that a ground truth fusion set is not available, and most studies depend on in silico simulation, a small number of synthetic fusions, and validation on a small set of fusions to test the performances of the algorithms. Neither of the aforementioned performance-testing strategies can be effectively used to comprehensively evaluate various fusion detection and filtering strategies and parameters. Here, we report a more sensitive computational algorithm ‘Somatic Fusions involving Non-Coding Sequences’ (SFyNCS) to detect fusions involving non-coding sequences. We used somatic SVs detected from whole-genome sequencing data to validate fusions detected from RNA-Seq data. This allowed us to find the best-performing fusion detection and filtering strategies. We then describe several recurrent and oncogenic fusions from 9565 TCGA tumor samples. The oncogenic function of one of the recurrent fusions involving non-coding sequences was validated in mouse model.

## MATERIALS AND METHODS

### SFyNCS algorithm

#### Identifying raw fusions

RNA-Seq reads were aligned by STAR (18) to the reference genome for detection of discordant read pairs and split reads. Discordant pairs were defined by STAR if they satisfied one of three conditions: paired-end reads aligned to different chromosomes, paired-end reads aligned to the same chromosome but in incompatible orientations, or paired-end reads in compatible orientations but with distances greater than 100 kb. Reads potentially spanning the fusion breakpoints that could not be aligned consecutively in the genome were split into two parts. If the two parts satisfied the same conditions above for discordant pairs, these reads were considered split reads. The 100 kb cutoff was used because the majority of introns are shorter than 100 kb (Supplementary Figure S1A), and 100 kb is longer than five folds of standard deviation of distances between two reads in pairs (Supplementary Figure S1B). Other algorithms, such as STAR-Fusion and Arriba, also used the same cutoff. Discordant pairs and split reads aligned to multiple locations were discarded and duplicated reads (read pairs with identical mapping) were removed. Discordant pairs and split reads were merged into clusters if they were aligned to the same chromosomes, had the same orientations and were within 1 Mb of each other. Raw fusions were then called from these clusters. Most genes are shorter than 1 Mb (Supplementary Figure S1C), so a 1 Mb cutoff was used to merge reads belonging to the same genes together. Note that the 1 Mb cutoff was very permissive and was intended to detect as many raw fusions as possible. Low quality fusions would be filtered out in later steps. Precise fusion breakpoints were determined by split reads. Split reads with the same orientation and within 5 bp were considered to support the same fusion. Each candidate fusion must be supported by at least one split read. In the initial detection phase, discordant read pair support was not required. Different numbers of read support (discordant read pair and split read) were tested in a later section. Note that one discordant pair may support more than one fusion (different isoforms) depending on how the transcripts were spliced (Supplementary Figure S2). Gene annotation was not used in raw fusion detection, so that fusion breakpoints in both protein-coding genes and non-coding regions of the genome could be detected. The process described above was very sensitive allowing a large number of raw fusions would be detected in each sample.

#### Testing filtering strategies

To detect high quality tumor-specific fusions, we comprehensively tested the performances of the fusion calling and filtering strategies as well as various cutoffs in two rounds. In the first round, we tested the following filters: (i) number of total read support (discordant pair and split read combined, cutoffs tested: ≥2, ≥3, ≥4 and ≥5); (ii) Number of split read support (cutoffs tested: ≥1, ≥2, ≥3, ≥4, ≥5); (iii) number of discordant pair support (cutoffs tested: 0 and ≥1); (iv) minimal distance between discordant pairs and split reads supporting the same fusion (≤100 bp, ≤200 bp, ≤500 bp, ≤1 kb, ≤5 kb, ≤10 kb, ≤20 kb, ≤50 kb, ≤100 kb, ≤200 kb, ≤300 kb, ≤500 kb, ≤1 Mb and NA [filter not applied]); (v) Whether or not to filter out deletion-like fusions that were within the same gene annotated by GENCODE; (vi) Whether or not to filter out duplication-like and inversion-like fusions that were within the same gene annotated by GENCODE; (vii) Fusion breakpoint distance for deletion-like fusions (produced by somatic deletions at the DNA level, cutoffs tested: ≥100 kb, ≥200 kb, ≥300 kb, ≥500 kb, ≥1 Mb and NA); (viii) Fusion breakpoint distance for duplication-like and inversion-like fusions (produced by somatic duplications and inversions at the DNA level, cutoffs tested: ≥10 kb, ≥20 kb, ≥30 kb, ≥50 kb, ≥100 kb, ≥200 kb, ≥300 kb, ≥500 kb, ≥1 Mb and NA); (ix) breakpoint flanking sequence identity by aligning 20 bp sequences (10 bp from both sides) of two breakpoints with Needleman–Wunsch algorithm (cutoffs tested: ≤0.3, ≤0.5, ≤0.8 and NA); (x) size of breakpoint flanking regions for filters (xi) and (xii) (cutoffs tested: 100 bp, 500 bp, 1 kb, 5 kb and 10 kb); (xi) standard deviation (SD) of fusion-supporting read clusters in fusion breakpoint flanking regions (described in detail in the next paragraph, cutoffs tested: ≥0.05, ≥0.1, ≥0.15, ≥0.2, ≥0.25, ≥0.3 and NA); (xii) number of fusion-supporting clusters in fusion breakpoint flanking regions (cutoffs tested: ≤5, ≤10, ≤15, ≤20, ≤25, ≤30 and NA); (xiii) Filtering by canonical splicing motifs (GT in the donor site, AAG/CAG/TAG in the acceptor site) within 5 bp of fusion breakpoints; (xiv) confirming discordant pair and split read alignment by TopHat2 (distance between TopHat2 and STAR alignments of split reads ≤5 bp); (xv) confirming split read alignment by BLAT and (xvi) filtering by fusion breakpoints detected in normal samples (more details below). Note that it is not practically feasible to test all combinations of different cutoffs. Therefore, only a selected subset were tested. In the second round, we either removed one filter, added one filter, or changed the cutoff for one filter based on the best performing filter combination determined in the first round. The two rounds of parameter search were performed iteratively until the best performing filters and cutoffs were found and no further improvement could be made (Supplementary Table S1).

For each candidate fusion breakpoint, there could be more than one read cluster supporting different fusions in its flanking region. Too many such clusters suggested that the read alignments of this region were unreliable. The number of fusion-supporting clusters was tested as a filtering strategy. Standard deviations (SDs) of the proportions of fusion-supporting reads in these clusters (equation below) was also tested.


\begin{eqnarray*} && {\mathrm{Standard \; deviation}}\;\left( {{\mathrm{SD}}} \right) = \sqrt {\frac{{\sum\nolimits_{i = 1}^N {{{\left( {{n}_i - \mu } \right)}}^2} }}{N}},\nonumber\\ && \qquad{\mathrm{where}}\;{n}_i = \frac{{{m}_i}}{{\sum\nolimits_{i = 1}^N {{m}_i} }} {\mathrm{and}}\;\mu = \frac{{\sum\nolimits_{i = 1}^N {{n}_i} }}{N}\end{eqnarray*}

*N* is the number of clusters, m_i_ is the number of reads in cluster *i*, *n_i_* is the proportion of reads in cluster *i*.

Normal samples from TCGA (Supplementary Table S2) were used to remove germline events and other systematic artifacts. A panel of 140 normal samples was first constructed by randomly selecting 10 normal samples from each tumor type that had more than 10 matched normal samples. Fusions detected in each tumor sample were filtered by this normal panel as well as all the matched normal samples of the corresponding tumor type when available. Note that some tumor types, such as lower-grade glioma and ovarian cancer, lacked matched normal samples. These tumor samples were solely filtered by the 140-sample normal panel. Fusions detected in tumor samples were discarded if there were at least two fusion supporting reads (either discordant read pairs or split reads) within 10 kb for both breakpoints in any normal samples.

Note that if the fusion breakpoints are located close to the end of the transcripts, discordant read pairs may not exist. Therefore, we tested the performance of fusion detection without the requirement of discordant read pair support. Since fusion breakpoints were determined by split reads, we did not test the performance of fusion detection without split read support.

The process of testing filtering strategies is very complex and time consuming, but it does not need to be done by the end users if they wish to use our recommended default parameters. For individual RNA-Seq samples, it would take 3 hours and 30 Gb of memory on average to call fusions using SFyNCS. Most of the run time and memory were used in aligning reads by STAR. Testing combinations of filters is an independent process and only necessary if the users wish to use different filters other than what we recommended. Since it is impossible to test all parameter combinations, we tested 166,178 combinations of filtering strategies and parameters. The performances of a subset of filtering strategies are provided in Supplementary Table S1, so that the users can choose other filters to increase sensitivity or precision based on their needs without repeating the entire testing process.

### Benchmarking fusion detection tools

Fusions in 338 TCGA samples were identified by Defuse (v0.8.1), FusionCatcher (v1.33), InFusion (v0.8.1-dev) and SQUID (v1.5) with default parameters. Note that SQUID failed to analyze TCGA-DX-A2IZ-01A-11R-A21T-07. Fusions detected by multiple tools needed to have identical breakpoint locations and orientations. Fusions were considered supported by somatic SVs if SV breakpoints could be found within 100 kb of fusion breakpoints and the DNA fragments produced by the SVs could be spliced into the corresponding fusion RNA. Fusions in MCF7 were identified by FusionCatcher (v1.33) with default parameters. Fusion-supporting split reads identified by both FusionCatcher (v1.33) and SFyNCS were aligned to the reference genome by BLAT to validate split-read alignment. If there were two segments of a split read aligned uniquely within 5 bp of the predicted fusion breakpoints, the split read was considered validated by BLAT. Split reads not validated by BLAT mainly belonged to the following three categories: (i) reads aligning entirely (more than 85 bp of 101 bp-long reads) to one location of the genome, (ii) one or both fusion breakpoints lacking support (i.e. not aligned within 5 bp of the predicted breakpoints) or (iii) reads aligning to multiple locations. If a fusion did not have any split read validated by BLAT, the fusion was considered not validated.

### Cell lines

HEK293T cells were obtained from Dr. Alexander Muir (University of Chicago). MCF7 cells were obtained from Dr. Lev Becker (University of Chicago). HCT116 and K562 cells were obtained from Dr Chuan He (University of Chicago). A549 cells were purchased from ATCC (American Type Culture Collection, USA). All cell lines were cultured at 37°C/5% CO_2_. HEK293T cells were cultured in Dulbecco's Modified Eagle Medium (DMEM) (Gibco, 21041025) supplemented with 10% fetal bovine serum (FBS), 1% penicillin/streptomycin and 2 mM l-glutamine. MCF7 cells were cultured in Eagle's Minimum Essential Medium (Corning, 10-010-CV) with 10% FBS (Gibco, A4766). HCT116 cells were cultured in McCoy's 5A Medium Modified (Gibco, 16600-082) with 10% FBS. K562 cells were cultured in Iscove's Modified Dulbecco's Medium (Gibco, 12440-053) with 10% FBS. A549 cells were cultured in F-12K Medium (ATCC, 30-2004) with 10% FBS and 1% penicillin/streptomycin. All cell lines have been regularly monitored and tested negative for mycoplasma using the mycoplasma detection kit (Lonza, LT07-218).

### RT-PCR and sanger sequencing validation

Twenty fusions were randomly selected for validation among the 238 fusions involving non-coding sequences (FiNCS) in MCF7 RNA-Seq data (19) detected by SFyNCS but not detected by FusionCatcher (v1.0), InFusion (v0.8), MapSplic2 (v2.2.1), SOAPfuse (v1.2.7), STAR-Fusion (v1.5.0) or EasyFuse (v1.3.0). Ten FiNCS detected in MCF7 RNA-Seq data produced by Cancer Cell Line Encyclopedia (CCLE) and Encyclopedia of DNA Elements (ENCODE) but not detected in the RNA-Seq data produced by the previous study (19) were randomly selected. Six FiNCS were randomly selected from HCT116 and K562 cell lines. Primers (Supplementary Table S3) were designed by Primer3 and synthesized by Integrated DNA Technologies. MCF7, HCT116 and K562 cells were plated in 6-well plates and allowed to reach 80% confluence prior to RNA extraction. After cells being lysed in 300 μl/well TRYzolTM (Invitrogen, 15596026), RNA samples were prepared following the manual of Direct-zol RNA Miniprep kit (RPI, ZR2052). Reverse transcription was performed using Applied Biosystems High-Capacity cDNA Reverse Transcription Kit (43-688-14) following manufacturer's instructions. PCR was conducted on SimpliAmpTM Thermo Cycler (Applied Biosystems, A24811), with HotStarTaq Plus Master Mix (QIAGEN, 1039620) following the manufacturer's instructions. PCR products were extracted from 2% agarose gel with MinElute Gel Extraction kit (QIAGEN, 28604) and purified with MinElute PCR purification kit (QIAGEN, 28004). Then the DNA samples were sent to the DNA Sequencing & Genotyping Facility of the University of Chicago Comprehensive Cancer Center for Sanger sequencing.

### Synthesis of *ZDHHC17-LNCKB.11978.4, mut-ZDHHC17-LNCKB.11978.4* and *RPSAP52-LNCKB.11978*

The 1870 bp *ZDHHC17-LNCKB.11978.4* wildtype and mutant (start codon mutated) fusion cDNAs and 1260 bp *RPSAP52*-*LNCKB.11978* fusion cDNA were synthesized by GenScript (New Jersey, USA) and subcloned into the lentiviral pCDH-CMV-MCS-EF1-Puro plasmid (SBI, CD510B-1). The cDNA sequences in the plasmid were verified by Sanger sequencing at University of Chicago Medicine Comprehensive Cancer Center core facility. The synthesized fusion sequences can be found in Supplementary Table S4.

### Lentiviral transduction and qPCR

The fusion sequences were subcloned into pCDH-CMV-Puro lentiviral vector and then co-transfected with psPAX2 and pMD2.G plasmids into HEK293T cells to generate lentiviral particles respectively. Empty pCDH-CMV-Puro lentiviral vector was also transfected as the control. After 48 hours, the lentivirus was harvested and transduced into A549 cells with 10 μg/ml polybrene. Puromycin (1 μg/mL) was added into cells at 48 hours post transduction for 7 days to establish stable A549 cell lines with fusions.

Total RNA from cells was isolated using Direct-zol RNA MiniPrep Kit (Zymo Research) according to the manufacturer's instructions. cDNA was synthesized using SuperScript VILO cDNA synthesis kit (Life Technologies). qPCR was performed using SYBR green qPCR Master Mix (Sigma) on an Applied Biosystems QuantStudio 3 Real-Time PCR System. Primer sequences used were as follows:

- *GAPDH* forward: 5′-GTCTCCTCTGACTTCAACAGCG-3′
- *GAPDH* reverse: 5′-ACCACCCTGTTGCTGTAGCCAA-3′
- *ACTIN* forward: 5′-CACCATTGGCAATGAGCGGTTC-3′
- *ACTIN* reverse: 5′-AGGTCTTTGCGGATGTCCACGT-3′
- *ZDHHC17-Inckb.11978* primer 1 forward: 5′-GAGTACGATACCGAAGCGGG-3′
- *ZDHHC17-Inckb.11978* primer 1 reverse: 5′-ACTGAGGTGAGGAGTGGGTT-3′
- *ZDHHC17-Inckb.11978* primer 2 forward: 5′-CGGCCCGGATGAGTACGATA-3′
- *ZDHHC17-Inckb.11978* primer 2 reverse: 5′-TAACGTTCACAGCACTCGGG-3′
- Mutant-*ZDHHC17*- *Inckb.11978* primer 1 forward: 5′-GAGTACGATACCGAAGCGGG-3′
- Mutant-*ZDHHC17*- *Inckb.11978* primer 1 reverse: 5′-ACTGAGGTGAGGAGTGGGTT-3′
- Mutant-*ZDHHC17*- *Inckb.11978* primer 2 forward: 5′-CGGCCCGGATGAGTACGATA-3′
- Mutant-*ZDHHC17*- *Inckb.11978* primer 2 reverse: 5′-TAACGTTCACAGCACTCGGG-3′
- *RPSAP*- *Inckb.11978* primer 1 forward: 5′-CTAGCACCAGTGGGCACATC-3′
- *RPSAP*- *Inckb.11978* primer 1 reverse: 5′-GTTCTGAGCAGGAGCATCGT-3′
- *RPSAP*- *Inckb.11978* primer 2 forward: 5′-TGGGCACATCGAGAGCAAAC-3′
- *RPSAP*- *Inckb.11978* primer 2 reverse: 5′-CAGAGGGAAGGGCTGATTCC-3′

### Xenograft models

NOD.CB17-Prkdc^scid^/J (NOD-SCID) mice were purchased from The Jackson Laboratory. All animal experiments complied with the standards approved by University of Chicago. For tumor transplantation, 5 × 10^5^ A549 cells with control and fusion vectors were resuspended in PBS and mixed with Matrigel (R&D Cultrex Type 3, Pathclear) at 1:1 ratio, followed by subcutaneously injection into NOD-SCID mice. Tumor volume was assessed by calipers every week. At 7 weeks post tumor grafting, animals were euthanized and the engrafted tumors were weighed and photographed.

## RESULTS

### SFyNCS overview

**Figure 1.**
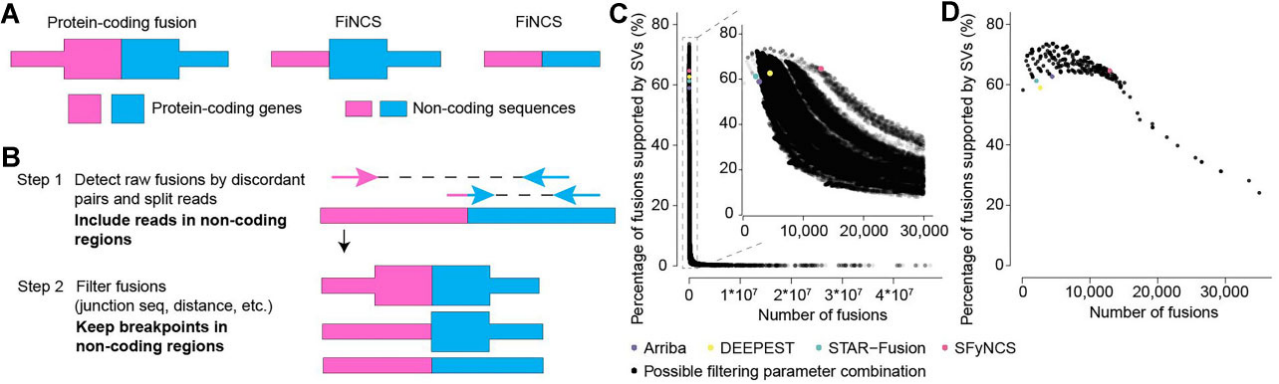
SFyNCS. (**A**) Fusions of different types. Pink and blue shapes denote two fusion partners. Fusions can be in any combination of protein-coding genes and non-coding sequences. (**B**) Overview of SFyNCS. There are two main steps: detect raw fusions and filter fusions. (**C**) A total of 166,178 combinations of filtering strategies and parameters are tested. Each dot represents one combination. The number of fusions is used to measure sensitivity, and the percentage of fusions supported by somatic SVs is used to measure specificity. A portion of the plot is zoomed in in the upper right corner. (**D**) Sensitivity and specificity of the final filtering strategy implemented in SFyNCS compared to changing one or a few parameters at a time. In both C and D, the sensitivity and specificity for Arriba, DEEPEST and STAR-Fusion are also shown.

**Figure 2.**
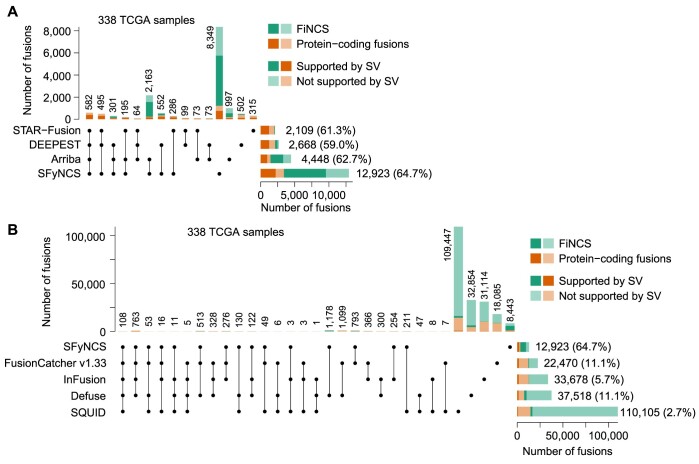
Benchmarking tools in TCGA samples. (**A**) UpSet plot of four fusion-detection algorithms in 338 TCGA samples with both WGS and RNA-Seq data. The stacked bars on the bottom right are the total fusions detected by four tools respectively. The stacked bars on the top show the number of fusions identified by one or more tools. The black dots under the stacked bars indicate tools used. The numbers on the top and on the right side of the bars are numbers of fusions. The percentages in the parentheses indicate percentages of fusions supported by somatic SVs. (**B**) Comparison of SFyNCS with four fusion-detection algorithms, FusionCatcher v1.33, InFusion, Defuse and SQUID, in the same 338 TCGA samples.

Here, we developed SFyNCS to detect both protein-coding and non-coding fusions from RNA-Seq data (Figure 1A). In this study, protein-coding fusions are defined as both fusion partners being protein-coding genes, whereas FiNCS have one or both fusion partners being non-coding sequences. We note that FiNCS may still encode proteins since the non-coding fusion partners may provide cryptic start or stop codons. SFyNCS searches for discordant read pairs and split reads, including those mapped to non-coding regions, to detect both protein-coding fusions and FiNCS (Figure 1B). We use very loose cutoffs to detect raw fusions — one split read support required to define fusion breakpoints (Methods). Therefore, in the detection phase, SFyNCS is very sensitive and a large number of raw fusions will be identified. Although many algorithms, such as STAR-Fusion (3) and Arriba (12), detect raw fusions similar to SFyNCS, the main advantage of SFyNCS lies in our search for the best performing filtering strategies (Methods). Since in silico simulations and synthetic fusions cannot fully mimic the artifacts and noise in real tumors, we sought to use fusions detected from real tumors to test fusion detection performances. Because ground truth fusions do not exist, to test performances, we took advantage of 338 tumor samples across 22 tumor types (Supplementary Table S5) with both RNA-Seq and whole-genome sequencing (WGS) data from TCGA cohort. Since tumor-specific fusions detected at the RNA level should be supported by somatic SVs detected at the DNA level, the 338 tumor samples allowed us to comprehensively evaluate different filtering strategies and cutoffs to determine the best performing filters. As it was not feasible to test all possible combinations of filtering strategies and cutoffs, we iteratively tested 166,178 combinations of cutoffs (Methods) until no further improvement could be made (Figure 1C, D and Supplementary Table S1). The final filters we chose to implement in SFyNCS with reasonable sensitivity and specificity were as follow: (i) at least one discordant read pair support; (ii) at least one split read support; (iii) at least three total read support (discordant read pair + split read); (iv) the minimal distance between the discordant pairs and the split reads to be ≤10 kb; (v) breakpoints for all intra-chromosomal fusions (deletion-like, duplication-like and inversion-like) not located in the same genes; (vi) fusion breakpoint distance for deletion-like fusions to be ≥500 kb; fusion breakpoint distance for duplication-like and inversion-like fusions to be ≥20 kb; (vii) standard deviation (SD) of fusion-supporting clusters within 100 bp of breakpoints to be ≥0.1; (viii) canonical splicing motif present within 5 bp of fusion breakpoints; (ix) not found in any normal samples. The detailed description of the filters can be found in Methods. Using these filters, SFyNCS detected 12,923 fusions in the 338 samples (Supplementary Table S6), 8356 (64.7%) of which were supported by somatic SVs (Figure 2A).

### Benchmarking SFyNCS

We compared SFyNCS with other algorithms in the same 338 samples from the previous section. Recently, STAR-Fusion (3), DEEPEST (16) and Arriba (12) reported 2109, 2668 and 4448 fusions in these samples, respectively (Figure 2A). In contrast, SFyNCS detected 12,923 fusions which were 6.1, 4.8 and 2.9 folds of the ones detected by STAR-Fusion, DEEPEST and Arriba, respectively. Therefore, the sensitivity of SFyNCS was far better than that of STAR-Fusion, DEEPEST and Arriba. The fractions of fusions supported by somatic SVs were quite similar across the four algorithms, ranging from 59.0% to 64.7% (Figure 2A). Fusions detected by SFyNCS had the highest SV support (64.7%). These metrics suggested that the quality of fusions detected by these four algorithms were quite similar, and the specificity of SFyNCS was slightly better than that of STAR-Fusion, DEEPEST and Arriba. Interestingly, in the 12,923 SFyNCS-detected fusions, 9520 (73.7%) were FiNCS and 64.7% of the FiNCS were supported by SVs. This suggested that the quality of FiNCS detected by SFyNCS was as good as the quality of protein-coding fusions. We further classified fusions based on the relative positions of the fusion partners (e.g. on the same chromosomes or not on the same chromosomes) and found that the quality of fusions in all categories was comparable (Supplementary Figure S3). STAR-Fusion and DEEPEST had limited ability in detecting FiNCS (Figure 2A). Arriba detected 2993 FiNCS, 2145 of which were also detected by SFyNCS. SFyNCS detected 8349 fusions that were missed by other algorithms. The vast majority (7135) of these were FiNCS. In addition, SFyNCS detected 1214 protein-coding fusions that were not detected by other algorithms. 63.3% of SFyNCS-specific fusions were supported by SVs, which suggested that they were of high quality. We then tested FusionCatcher (20), InFusion (21), Defuse (22) and SQUID (23) on the 338 tumors (Supplementary Table S6). These four algorithms detected many more fusions than SFyNCS, ranging from 22,470 to 110,105 (Figure 2B). However, the fractions of fusions supported by SVs for these four algorithms ranged from 2.7% to 11.1% (Figure 2B) indicating that the majority of these fusions were false calls. This suggested that the specificity of SFyNCS was far better than FusionCatcher, InFusion, Defuse and SQUID.

**Figure 3.**
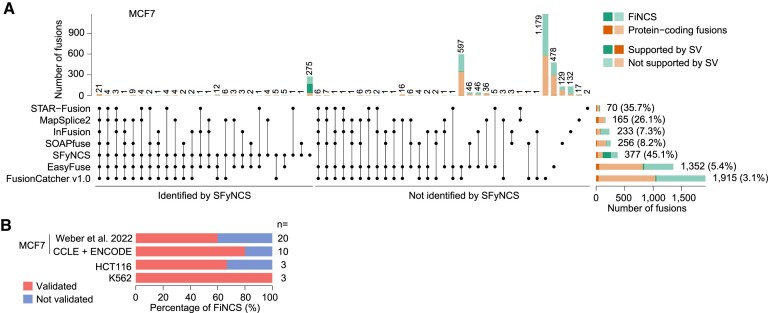
Benchmarking tools in MCF7 cell line. (**A**) Comparison of SFyNCS with six fusion detection algorithms in MCF7 cell line: STAR-Fusion, MapSplice2, InFusion, SOAPfuse, EasyFuse and FusionCatcher v1.0. Stacked bars on top are grouped into fusions identified by SFyNCS and not identified by SFyNCS. The stacked bars on the bottom right are the total fusions detected by seven tools respectively. The stacked bars on the top show the number of fusions identified by one or more tools. The black dots under the stacked bars indicate tools used. The numbers on the top and on the right side of the bars are numbers of fusions. The percentages in the parentheses indicate percentages of fusions supported by somatic SVs. (**B**) Percentages of FiNCS validated by PCR and Sanger sequencing in three cancer cell lines. The number of FiNCS tested is shown on the right side of bars.

We further tested SFyNCS on the breast cancer cell line MCF7 and compared it to six algorithms that were previously tested (19) on MCF7 (STAR-Fusion, MapSplice2 (24), InFusion, SOAPfuse (25), FusionCatcher and EasyFuse (19)). SFyNCS detected a total of 377 fusions, including 262 (69.5%) FiNCS (Figure 3A and Supplementary Table S7). In SFyNCS-detected fusions, 45.1% of the fusions were supported by SVs. STAR-Fusion, MapSplice2, InFusion and SOAPfuse detected fewer fusions than SFyNCS (ranging from 70 to 256) and the fractions of fusions supported by SVs were lower than SFyNCS (ranging from 7.3% to 35.7%) (Figure 3A). EasyFuse and FusionCatcher detected many more fusions (1352 and 1915 respectively). However, very few of them were supported by SVs (5.4% and 3.1% respectively) (Figure 3A). In order to validate the fusions predicted by FusionCatcher, we extracted split reads provided by FusionCatcher and aligned them to the reference genome by BLAT. We found that only 16.5% of the fusions predicted by FusionCatcher were supported by the split reads, which was in sharp contrast to SFyNCS (80.6%) (Supplementary Figure S4A–E). This suggested that the majority of fusions detected by FusionCatcher were likely false positives due to alignment errors. EasyFuse used 5 algorithms to detect fusions: STAR-Fusion, MapSplice2, InFusion, SOAPfuse and FusionCatcher. FusionCatcher was the only one detected a large number of fusions (Figure 3A). Therefore, EasyFuse likely suffered from similar alignment errors. Among all these algorithms, only STAR-Fusion had comparable specificity to SFyNCS, but it detected five-fold fewer fusions than SFyNCS. SFyNCS detected 275 fusions that were not detected by any other algorithm in MCF7, including 238 FiNCS. In the 275 SFyNCS-specific fusions, 49.1% were supported by SVs (Figure 3A), which suggested that SFyNCS-specific fusions were of high quality. We randomly selected 20 FiNCS detected only by SFyNCS, performed PCR and Sanger sequencing validation and were able to validate 12 (60%) of them (Figure 3B, Supplementary Figure S5 and Supplementary Table S3). We further detected fusions in the MCF7 cell line using different RNA-Seq data produced by CCLE and ENCODE and found an additional 237 fusions (Supplementary Figure S4F and Supplementary Table S7). We then randomly selected 10 FiNCS detected only in CCLE and ENCODE data and were able to validate 8 (80%) of them (Figure 3B, Supplementary Figure S6 and Supplementary Table S3). Moreover, we validated 5 out of 6 (83%) randomly selected FiNCS in the colorectal cancer cell line HCT116 and the leukemia cell line K562 (Figure 3B, Supplementary Figure S7, Supplementary Tables S3, S8 and S9).

Taken together, SFyNCS can detect many more fusions with better specificity than other existing algorithms, and the FiNCS detected by SFyNCS are highly accurate.

### Fusion landscape in TCGA cohort

**Figure 4.**
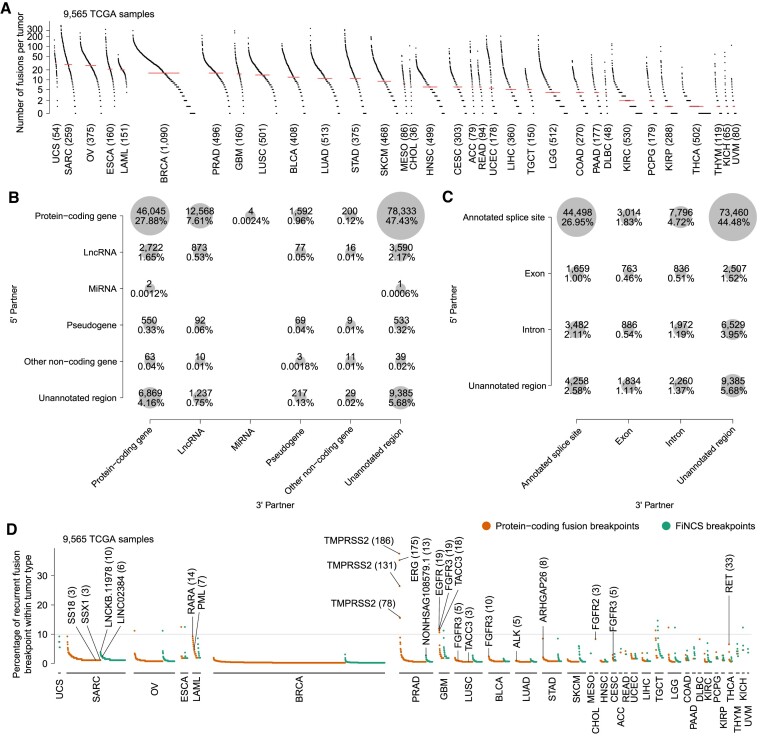
The landscape of fusion and recurrent fusion breakpoint in TCGA samples. (**A**) The landscape of fusions in 9565 TCGA samples. Each dot represents a tumor sample grouped by tumor type. Tumor types are sorted by median number of fusions per sample which is indicated by the red lines. The numbers in the parentheses are the numbers of tumor samples in the corresponding tumor types. (**B** and **C**) Classifications of fusion partners. The 5′ and 3′ fusion partners are shown as y and x axes. The size of each circle represents the number of fusions in the corresponding category. (**D**) Recurrent fusion breakpoints in 9565 TCGA samples. Each orange or green dot represents a recurrent fusion breakpoint detected in at least three samples. The y axis indicates the percentage of samples carrying the fusion breakpoints in the corresponding tumor types. The numbers in parentheses represent numbers of samples carrying the breakpoints. All breakpoints are at base-pair level. For example, *TMPRSS2*-*ERG* is the most recurrent fusion in adult solid tumors and can be detected in 183 out of 496 prostate cancers. Among them, 168 tumors have more than one *TMPRSS2*-*ERG* isoform involving various exons of *TMPRSS2*. Therefore, 3 out of the top 4 recurrent fusion breakpoints in prostate cancer are in *TMPRSS2* gene and these breakpoints are observed in 186, 131 and 78 samples.

We then used SFyNCS to analyze 9565 TCGA tumor samples from 33 tumor types (Supplementary Table S5). A total of 165,139 fusions were detected (Supplementary Table S10). Intriguingly, 119,191 (72.2%) of the fusions were FiNCS and were much more abundant than protein-coding fusions. Each tumor carried a median of 7 fusions ranging from 0 to 426 per tumor (Supplementary Table S11). Uterine Carcinosarcoma (UCS) and sarcoma (SARC) were the most abundant in fusions with medians of 32 and 29, respectively, whereas most kidney chromophobe cancers (KICH) and uveal melanomas (UVM) had less than 3 fusions (Figure 4A). The abundance of fusions was consistent with somatic SV frequencies across tumor types (26). STAR-Fusion, DEEPEST and Arriba detected far fewer fusions in TCGA samples (25,664, 31,007 and 48,545, respectively) (3,12,16). We further classified fusion partners detected by SFyNCS into protein-coding genes, long non-coding RNAs (lncRNAs), microRNAs (miRNA), pseudogenes, other non-coding genes and unannotated regions. Most fusions were protein-coding genes fused to unannotated regions (Figure 4B). In addition, we classified the fusion breakpoints into annotated splice sites, within exons, within introns and unannotated regions. Most fusions were annotated splice sites fused to unannotated regions (Figure 4C).

SFyNCS detected all known oncogenic fusions reported in these samples (3) (Figure 4D), such as *TMPRSS2*-*ERG*, *FGFR3*-*TACC3* and *PML*-*RARA*. To better identify candidate driver FiNCS, we relied on recurrent fusion breakpoints at base-pair level since the annotation of non-coding genes remains incomplete. At the base-pair level, there were a total of 1128 recurrent (occurring in at least 3 samples within the corresponding tumor type) fusion breakpoints involving non-coding sequences (Figure 4D, Supplementary Table S12). Interestingly, except for prostate cancer (PRAD), the most recurrent fusion breakpoints involving non-coding sequences were often as frequent as protein-coding fusion breakpoints in many tumor types (Figure 4D).

### Recurrent driver fusions involving non-coding sequences

**Figure 5.**
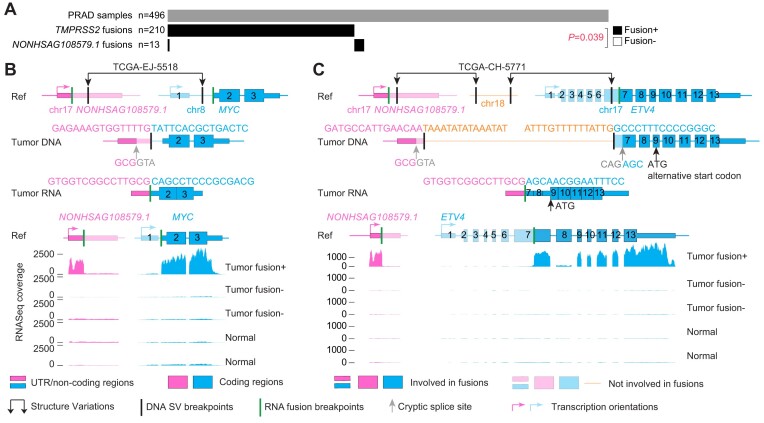
Recurrent FiNCS in prostate cancer. (**A**) Oncoprint plot of 496 prostate cancers showing fusions involving *TMPRSS2* and *NONHSAG108579.1*. (**B** and **C**) Structures of two *NONHSAG108579.1* fusions and their expression. The top three rows are gene and fusion structure cartoons of the reference genome, tumor DNA and tumor RNA. Pink and blue boxes denote two fusion partners. The *NONHSAG108579.1*-*ETV4* fusion in sample TCGA-CH-5771 is produced by two different translocations. The orange fragment from chromosome 18 is entirely spliced out from the fusion transcript. Five tracks of RNA-Seq coverage are shown for five samples at the bottom and the reference gene structures are given above the five tracks. Exons and introns are re-scaled to better illustrate fusion structures. In (B), the tumor samples without fusions (fusion-) are TCGA-HI-7169-01A-11R-2118-07 and TCGA-EJ-A7NJ-01A-22R-A352-07, and the normal samples are TCGA-EJ-7327-11A-01R-2118-07 and TCGA-HC-7742-11A-01R-2118-07. In (C), the fusion-samples are TCGA-G9-6365-01A-11R-1789-07 and TCGA-HI-7169-01A-11R-2118-07, and the normal samples are TCGA-EJ-7123-11A-01R-1965-07 and TCGA-EJ-7125-11A-01R-1965-07.

In 496 prostate cancers, we identified 27 FiNCS in 13 samples (2.6%) involving a long non-coding RNA (lncRNA) *NONHSAG108579.1* on chromosome 17. *NONHSAG108579.1* is expressed in several tissues including prostate, stomach, lung and pancreas (Supplementary Figure S8). The transcription start site of *NONHSAG108579.1* has strong H3K27ac signals in both a prostate cancer cell line and normal prostate gland (Supplementary Figure S9). This lncRNA acted as the 5′ fusion partner (Supplementary Table S13). These FiNCS were mutually exclusive with the well-known ETS fusions (*P* = 0.039, one-sided Fisher's exact test, Figure 5A). Two out of the 13 samples had WGS data, and in both samples, somatic translocations at the DNA level supported the FiNCS (Figure 5B and C). In sample TCGA-EJ-5518, there was a somatic translocation between chromosomes 8 and 17 (Figure 5B). The translocation brought *NONHSAG108579.1* and *MYC* together to produce a chimeric transcript. Exons 2 and 3 of *MYC* were fused with *NONHSAG108579.1* and the chimeric transcript could produce an intact MYC protein (Figure 5B). In sample TCGA-CH-5771, there were two somatic translocations involving chromosomes 17 and 18 resulting in *NONHSAG108579.1* being fused to *ETV4* with an 8.9kb fragment from chromosome 18 inserted in-between (Figure 5C). At the RNA level, the chromosome 18 fragment was entirely spliced out. On exon 9 of *ETV4*, there was an alternative start codon, and therefore, the *NONHSAG108579.1*-*ETV4* fusion transcript could produce a short ETV4 protein. In all *NONHSAG108579.1* fusions, the 3′ fusion partners lost their promoters and the fusion transcripts were transcribed from the *NONHSAG108579.1* promoter (Figure 5B, C and Supplementary Figure S10). Therefore, these fusions could be considered cases of promoter swapping. Two fusions could produce wildtype proteins (Figure 5B and Supplementary Figure S10G), whereas most of the fusions produced truncated proteins (Figure 5C, Supplementary Figure S10A–F and H). The lncRNA *NONHSAG108579.1* was expressed at low levels in normal prostate tissues and fusion-negative prostate cancers, but highly expressed in most fusion-positive tumor samples (Figure 5B, C and Supplementary Figure S10). Most of the 3′ fusion partners were activated (Figure 5B and C) and had expression patterns consistent with known driver fusions (27), characterizing by higher read coverage in exons included in the fusion transcripts than exons absent from the fusion transcripts. Furthermore, many of the 3′ fusion partners were well-known oncogenes including *MYC*, *ETV4*, *ETV1* and *BRAF* (Supplementary Table S13). Therefore, the *NONHSAG108579.1* fusions in prostate cancers were highly likely to be oncogenic.

We then compared multiple tools for their ability to detect driver fusions in PRAD. In the 496 tumors, SFyNCS detected 210 *TMPRSS2* fusions and 13 *NONHSAG108579.1* fusions (Supplementary Figure S11). Arriba, DEEPEST and STAR-Fusion detected fewer *TMPRSS2* fusions and *NONHSAG108579.1* fusions than SFyNCS (Supplementary Figure S11). Both DEEPEST and STAR-Fusion failed to detect any *NONHSAG108579.1* fusions. Although Arriba detected 8 *NONHSAG108579.1* fusions, it only detected 149 *TMPRSS2* fusions which was far fewer than SFyNCS, DEEPEST and STAR-Fusion. Therefore, SFyNCS is the most sensitive algorithm for both protein-coding fusions and FiNCSs.

**Figure 6.**
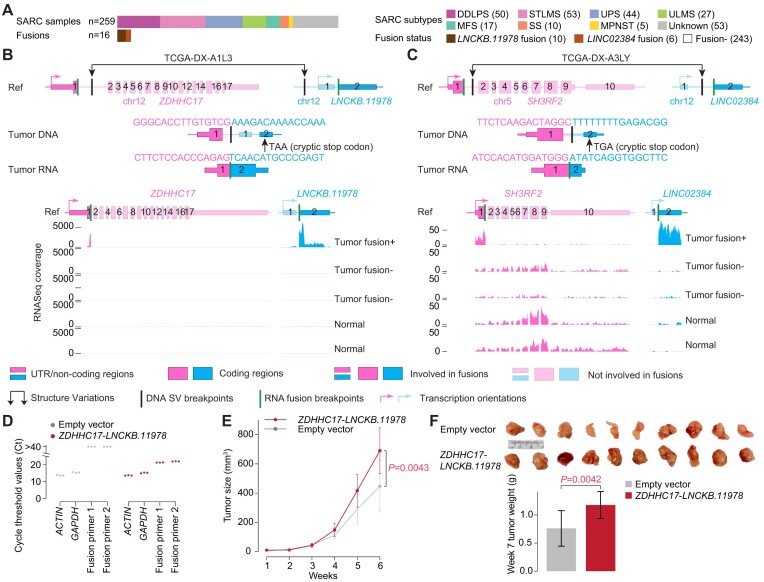
Recurrent FiNCS in sarcoma. (**A**) Oncoprint plot of 259 sarcomas showing FiNCS involving *LNCKB.11978* and *LINC02384*. DDLPS: dedifferentiated liposarcoma, STLMS: Soft Tissue Leiomyosarcoma, UPS: Undifferentiated Pleomorphic Sarcoma, ULMS: Gynecologic Leiomyosarcoma, MFS: Myxofibrosarcoma, SS: Synovial Sarcoma, MPNST: Malignant Peripheral Nerve Sheath Tumor. (**B** and **C**) Structures of a *LNCKB.11978* fusion and a *LINC02384* fusion in DDLPS and their expression. The top three rows are gene and fusion structure cartoons of the reference genome, tumor DNA and tumor RNA. Pink and blue boxes denote two fusion partners. The tumor samples without fusions (fusion-) are TCGA-IE-A4EI-01A-11R-A24X-07 and TCGA-IW-A3M4-01A-11R-A21T-07, and the normal samples are SRX636240 and SRX640265, respectively. (**D**) Quantitative PCR showing the presence of *ZDHHC17*-*LNCKB.11978* fusion transcript in A549 cells. (**E**) Tumor growth curves after subcutaneous injection from week 1 to week 6. Error bars are standard deviations. *P* value is calculated by two-sided Student's *t*-test. (**F**) Pictures of 10 tumors and tumor weights at week 7 after subcutaneous injection. Error bars are standard deviations. *P* value is calculated by two-sided Student's t-test.

In addition, recurrent FiNCS involving two lncRNAs (*LINC02384* and *LNCKB.11978*) were detected in 259 sarcomas (Supplementary Table S14). All of these FiNCS were detected in dedifferentiated liposarcomas (DDLPS), but not in other subtypes, and they were mutually exclusive with each other (Figure 6A). *LINC02384* and *LNCKB.11978* fusions occurred in 6 (12%) and 10 (20%) DDLPS tumors, respectively, and both lncRNAs were the 3′ fusion partners. The 5′ fusion partners were either protein-coding genes, lncRNAs or pseudogenes (Supplementary Table S14). Among the 16 fusion-positive tumors, 6 had WGS data and somatic SVs at the DNA level supported the FiNCS in all six samples (Figure 6B, C, Supplementary Figures S12 and S13). In sample TCGA-DX-A1L3, a somatic tandem duplication was present in protein-coding gene *ZDHHC17* and upstream of *LNCKB.11978* (Figure 6B). Exon 1 of *LNCKB.11978* was skipped and a chimeric transcript of exon 1 of *ZDHHC17* and exon 2 of *LNCKB.11978* was produced. The transcript could be translated into *LNCKB.11978* and produced a chimeric protein (Figure 6B). In sample TCGA-DX-A3LY, there was a somatic translocation between chromosomes 5 and 12 (Figure 6C). Similarly, a transcript of exon 1 of *SH3RF2* and exon 2 of *LINC02384* was produced and could be translated into a chimeric protein (Figure 6C). In most of these FiNCS involving *LNCKB.11978* and *LINC02384*, the 3′ lncRNAs were activated (Figure 6B, C, Supplementary Figures S12 and S13). The high recurrence and expression patterns indicated that these FiNCS were potential cancer drivers. To test the oncogenic functions experimentally, we synthesized the *ZDHHC17*-*LNCKB.11978* fusion, transduced it into A549 cells (Figure 6D), and injected the cells into immune deficient mice subcutaneously. Although the cancer cells did not grow differently in culture, tumors carrying the fusion grew significantly faster than controls (Figure 6E and F) upon grafting on mice, suggesting that the *ZDHHC17*-*LNCKB.11978* fusion does indeed have oncogenic activity. To further test whether the oncogenic function of the *ZDHHC17*-*LNCKB.11978* fusion, which was capable of producing a chimeric protein (Figure 6B), was mediated by protein or RNA, we synthesized two additional fusion constructs: mut-*ZDHHC17*-*LNCKB.11978* and *RPSAP52*-*LNCKB.11978*. Mut-*ZDHHC17*-*LNCKB.11978* had the exact same sequence as *ZDHHC17*-*LNCKB.11978* fusion but its start codon was mutated so that mut-*ZDHHC17*-*LNCKB.11978* did not have any open reading frames (ORFs). *RPSAP52* is a pseudo gene and was fused to *LNCKB.11978* in tumor TCGA-DX-AB2S (Supplementary Table S14). The *RPSAP52*-*LNCKB.11978* fusion did not encode any ORFs either. Upon engrafting mice, both mut-*ZDHHC17*-*LNCKB.11978* and *RPSAP52*-*LNCKB.11978* promoted *in vivo* tumor growth (Supplementary Figure S14), although not reaching statistical significance due to large variations in animal experiments. These results suggested that *LNCKB.11978* fusions are likely oncogenic at the RNA level.

Taken together, our results demonstrate that SFyNCS is able to detect oncogenic fusions involving non-coding sequences.

## DISCUSSION

Here, we describe our fusion detection algorithm SFyNCS which can detect fusions of both protein-coding genes and non-coding sequences in transcriptome sequencing data. SFyNCS is designed for Illumina short-read sequencing data and will suffer from the limitations of short-read sequencing technology, such as the lack of ability to resolve repetitive regions in the highly repetitive human genome. Fusion breakpoints in transposable elements, segmental duplications, satellite repeats, simple repeats and other types of repeats are unlikely to be reliably detected. This constraint is not specific to SFyNCS. All short-read based fusion detection algorithms suffer from this limitation. We note that fusions not supported by somatic SVs may still be true fusions, since SV breakpoints may not be identified in repetitive regions and the corresponding fusion breakpoints are in unique mappable regions. In addition, some fusions may be subclonal and the supporting SVs may not have enough sequencing coverage to be detected.

Another obstacle is the availability of normal samples to filter out germline events and systematic artifacts. Several tumor types do not have RNA-Seq data from matched normal samples, such as acute myeloid leukemia (LAML), lower grade glioma (LGG), ovarian cancer (OV), testicular germ cell tumors (TCGT) and uterine carcinosarcoma (USC). Some tumor types have very few matched normal samples, such as esophageal cancer (ESCA), glioblastoma (GBM), skin cutaneous melanoma (SKCM) and thymoma (THYM). Therefore, many of the highly recurrent fusions detected from these tumor types are likely not cancer drivers.

Although SFyNCS displayed superior performances in our benchmarking tests compared to existing tools, a small fraction of true fusions were still missed by SFyNCS. Each filter we implemented may remove some true fusions; for example, true fusion junctions may not always be canonical splice sites (27). For other types of somatic variants, including single nucleotide variants (SNVs), copy number variations (CNVs) and SVs, multiple tools are often integrated together for variant calling (28). Therefore, we recommend that users apply multiple tools to perform comprehensive fusion detection.

Mutual exclusivity has been used to infer driver genes altered by somatic SNVs and CNVs (29–31). A recent study on fusions in pediatric cancers applied mutual exclusivity to infer driver fusions (32). In our study, the FiNCS we detected in both prostate cancers and sarcomas were either mutually exclusive with known driver fusions (Figure 5A), or mutually exclusive with each other (Figure 6A). Such mutual exclusivities provided strong evidence that these FiNCS are likely driver fusions.

## Supplementary Material

gkad705_Supplemental_Files

## Data Availability

RNA-Seq data for 9565 tumor and 715 normal samples from TCGA (Supplementary Table S5) were downloaded from Genomic Data Commons (https://portal.gdc.cancer.gov/). RNA-Seq data for MCF7, HCT116 and K562 cell lines were downloaded from the National Center for Biotechnology Information (NCBI) Sequence Read Archive (SRA) with accession SRX5414642 (MCF7, CCLE), SRX159831 (MCF7, ENCODE), SRX6378523 (MCF7 Weber et al.), SRX6378524 (MCF7 Weber et al.), SRX5414471 (HCT116, CCLE) and SRX159835 (HCT116, ENCODE), SRX5414683 (K562, CCLE), SRX1603406 (K562, ENCODE) and SRX1603407 (K562, ENCODE). RNA-Seq data for two normal adipose tissue samples (SRX636240, SRX640265) from Genotype-Tissue Expression (GTEx) were downloaded from NCBI SRA. The H3K27ac ChIP-Seq signals for PC-3 cell line (ENCFF224GSO) and prostate gland (ENCFF143LGC) were downloaded from ENCODE portal (https://www.encodeproject.org/). The GTEx RNA-Seq read coverage in the region of NONHSAG108579.1 was downloaded from UCSC (https://genome.ucsc.edu/). Somatic SVs in TCGA samples were obtained from a recent Pan-cancer Analysis of Whole Genomes (PCAWG) study (26). Somatic SVs in MCF7 were downloaded from the Dependency Map (DepMap) portal (https://depmap.org/portal/). Fusions in TCGA samples identified by Arriba, DEEPEST and STAR-Fusion were downloaded from the related publications (3,12,16). Fusions in MCF7 identified by FusionCatcher (v1.0), InFusion (v0.8), MapSplic2 (v2.2.1), SOAPfuse (v1.2.7) and STAR-Fusion (v1.5.0) were downloaded from the previous study (19). Fusions in MCF7 identified by EasyFuse (v1.3.0) were provided by Dr. Ugur Sahin. The subtypes of sarcomas were obtained from a previous study (33). All coordinates were based on hg38 reference genome. GENCODE v29 was used for gene annotation. NOCODE v6 and lncRNAKB v7 were used to annotate non-coding genes that are not annotated by GENOCDE. The SFyNCS package is available at https://github.com/yanglab-computationalgenomics/SFyNCS (permanent DOI 10.5281/zenodo.8222797).
