# Supplementary material for: SFyNCS detects oncogenic fusions involving non-coding sequences in cancer
Source: Nucleic Acids Res. 2023 Aug 28;51(18):e96. doi: 10.1093/nar/gkad705 (PMC10570049; doi:10.1093/nar/gkad705)
Supplement: gkad705_Supplemental_Files [file gkad705_supplemental_files.zip › SFyNCS.supplementary.figures.docx]

**SFyNCS detects oncogenic fusions involving non-coding sequences in cancer**

**Supplementary Figures**


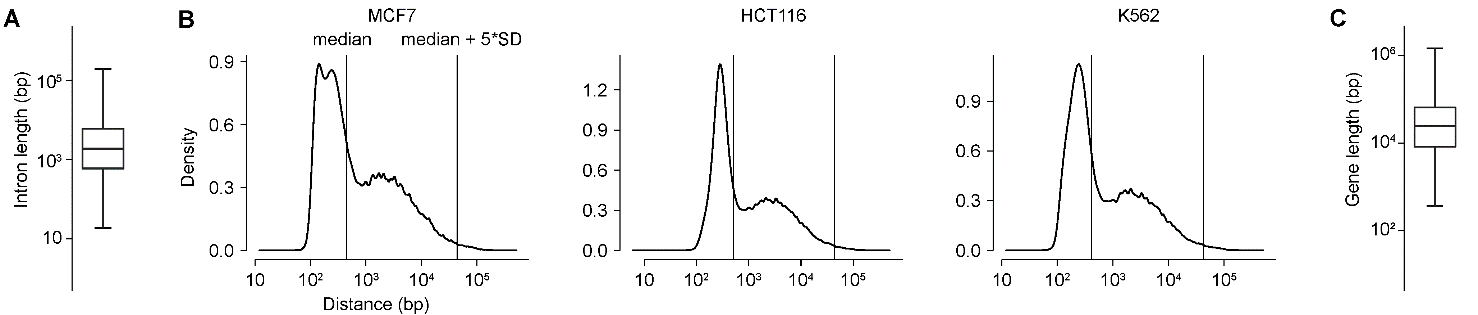


**Figure S1. Distributions of intron length, distance between read pairs and gene length.** **(A)** Distribution of intron length based on gene annotation of GENCODE v29. The box shows median, first and third quartiles, respectively. The whiskers extend at the 1.5 interquartile range. **(B)** Distributions of distances between two reads in pairs for RNA-Seq data of MCF7, HCT116 and K562. The two vertical lines represent median and median + 5 * standard deviation. Read pairs with distances more than 100 kb are considered abnormal (discordant read pairs). **(C)** Distribution of gene length based on gene annotation of GENCODE v29.


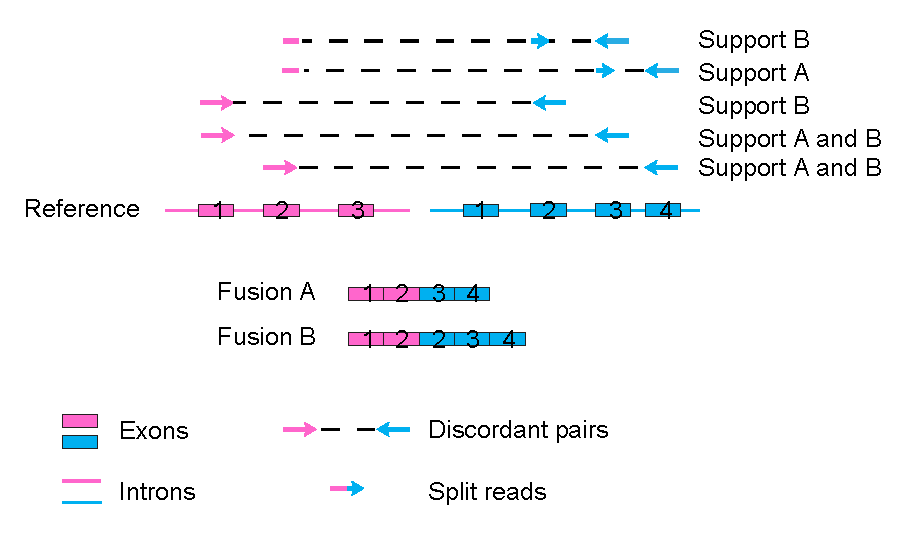


**Figure S2. Scheme of discordant read pairs and split reads supporting different isoforms.** Two isoforms are produced from the fusion locus. Some discordant pairs can support multiple isoforms.


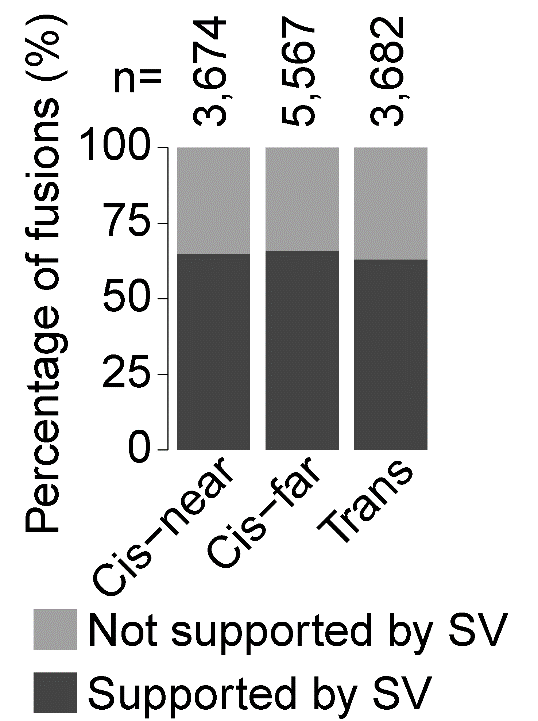


**Figure S3.** **Classification of fusions based on relative locations of fusion partners.** Two fusion partners located on the same chromosome are considered “cis”, whereas two partners on different chromosomes are “trans”. Cis-near are fusions with distances between two breakpoints being less than 1 Mb, whereas cis-far are fusions with distances between two breakpoints being more than 1 Mb.


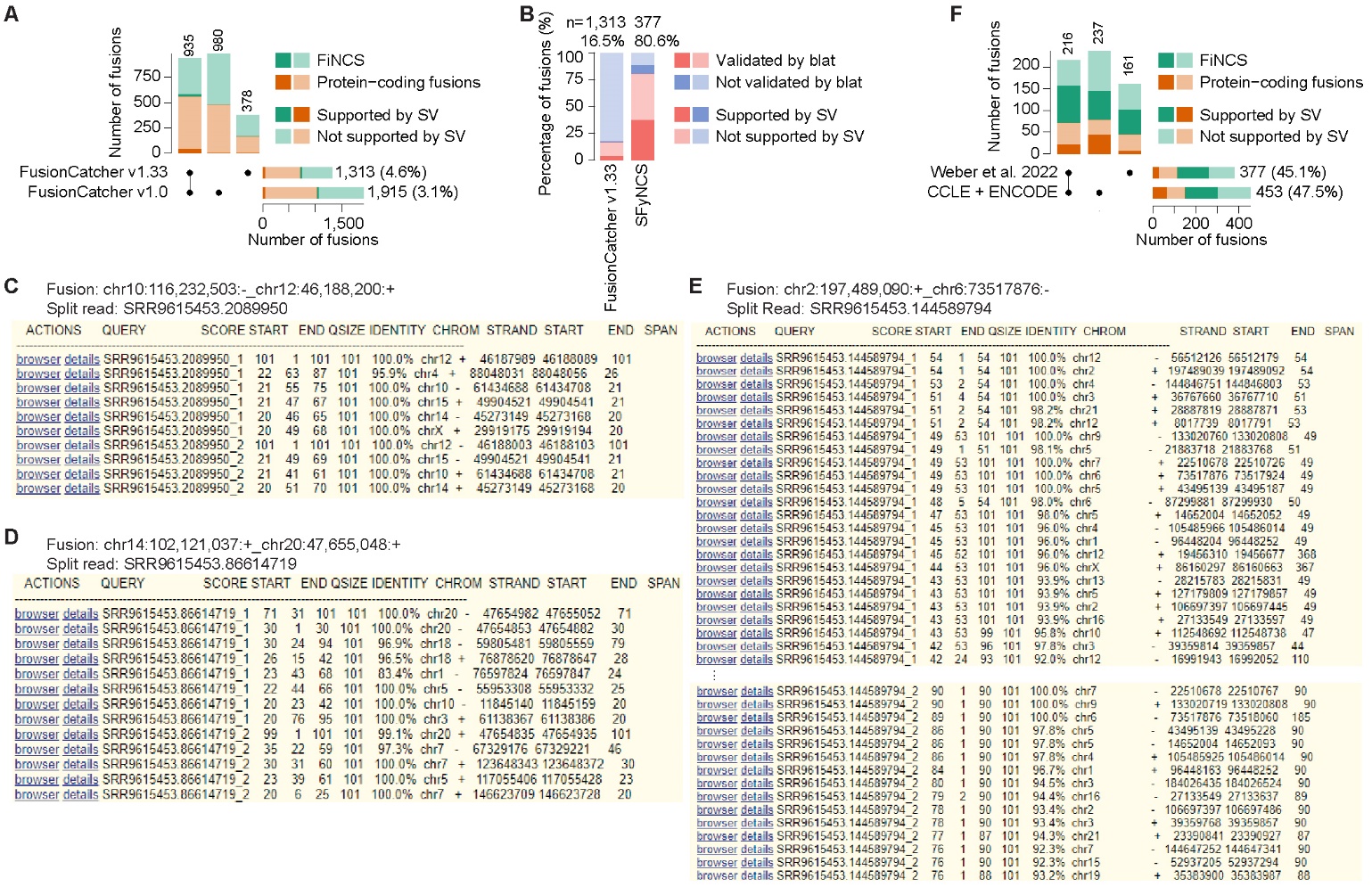


**Figure S4. Benchmarking fusion detection in MCF7 cell line.** (**A**) Comparison between FusionCatcher v1.0 and v1.33. The stacked bars on the top show the number of fusions identified by one or both versions. The stacked bars on the bottom right are the total fusions detected by the two versions. The black dots under the stacked bars indicate versions used. The numbers on the top and on the right side of the bars are numbers of fusions. The percentages in the parentheses indicate percentages of fusions supported by somatic SVs. (**B**) Barplot showing re-alignments of split reads by BLAT for fusions detected by FusionCatcher v1.33 and SFyNCS. (**C**, **D** and **E**) Examples of BLAT alignments that do not support the predicted fusions. Coordinates of predicted fusions and read names of split reads are listed on the top. BLAT screenshots are provided for split reads aligned to the hg38 reference genome. C, The split read can be aligned entirely to one location of the genome. D, The split read only supports one fusion breakpoint (chr20:47,655,048:+) but not the other (chr14:102,121,037:+). E, The split read can be aligned to many genomic regions. There are 105 alignments provided by BLAT and only 39 are shown. (**F**) Comparison between fusions detected by SFyNCS from RNA-Seq data produced by Weber et al. 2022, as well as CCLE and ENCODE.


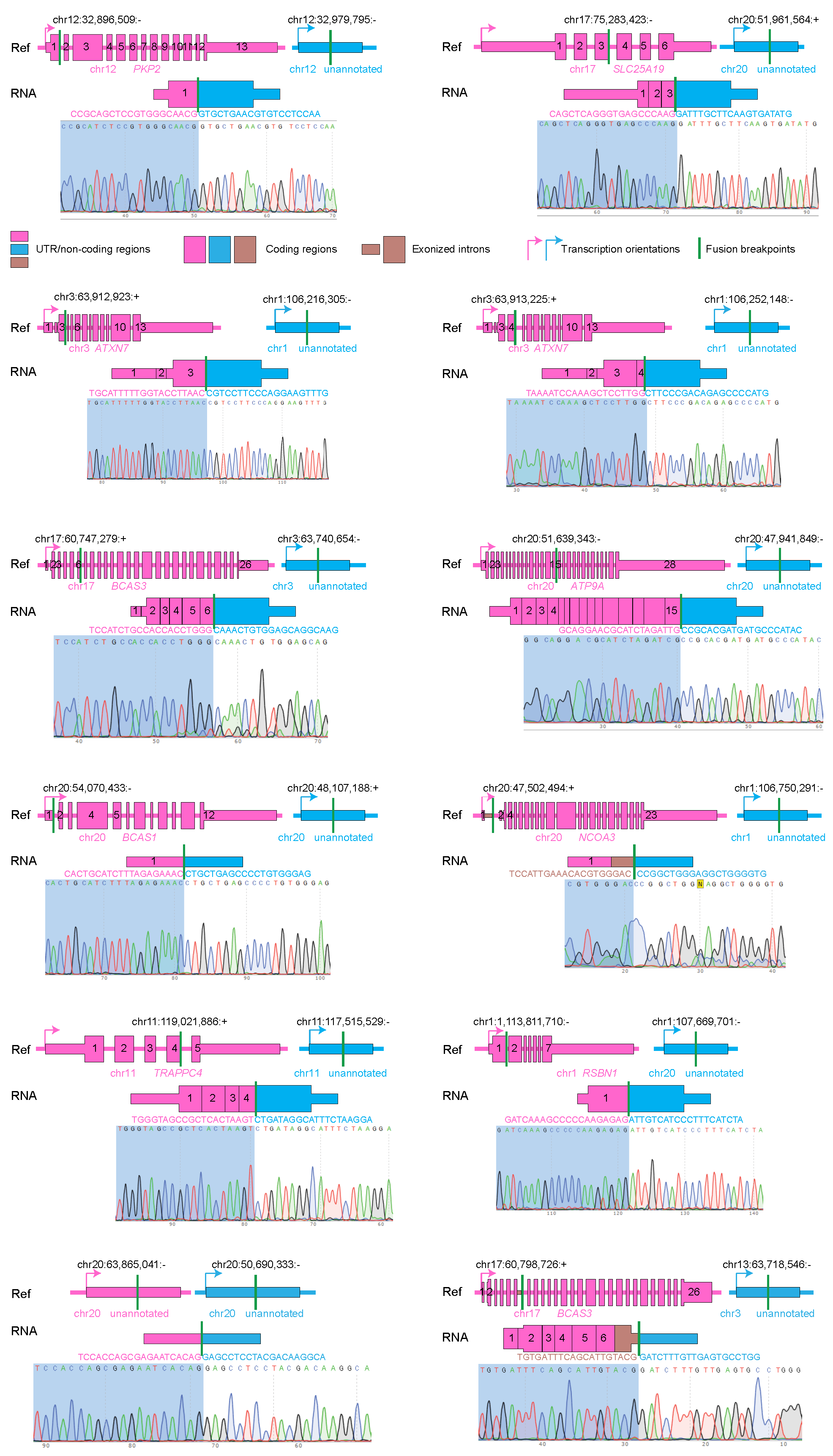


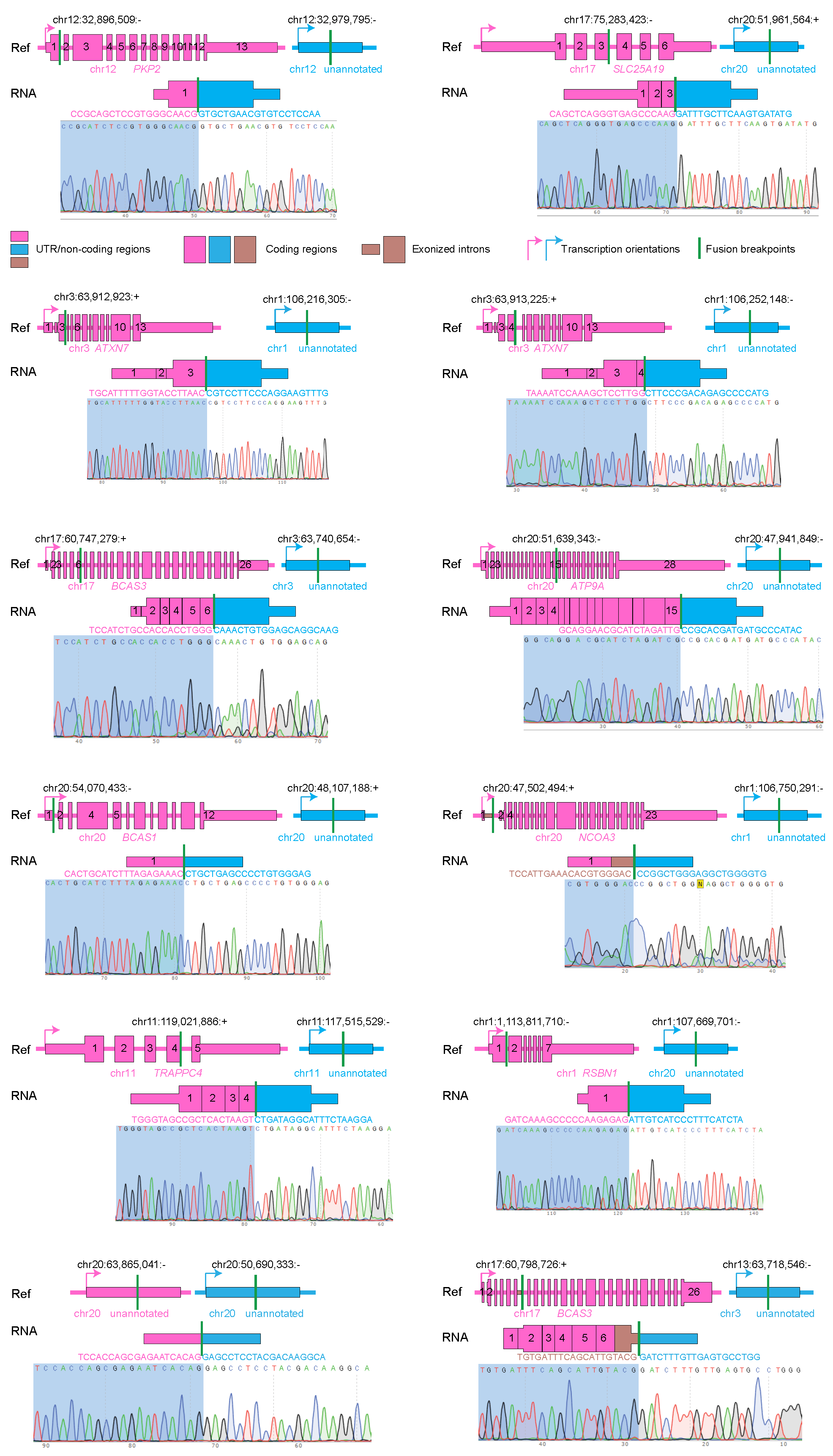


**Figure S5. Sanger sequencing validation for FiNCS in cell line MCF7 only detected by SFyNCS, but not by STAR-Fusion, MapSplice2, InFusion, SOAPfuse, EasyFuse or FusionCatcher.** Gene structures are shown in the reference tracks and fusion structures are shown in the fusion tracks. Sequences at the fusion breakpoints are shown above the Sanger sequencing chromatograms.


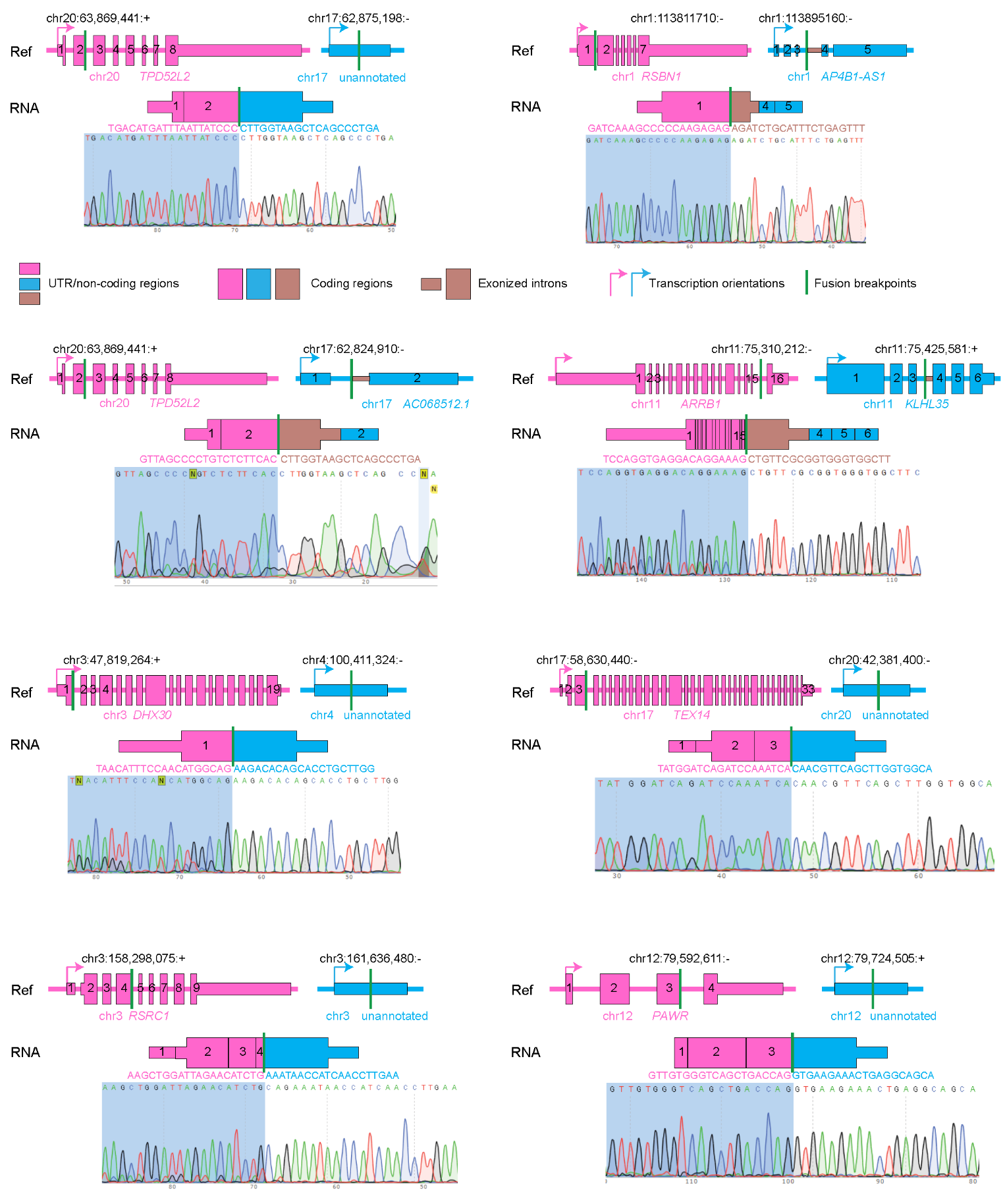


**Figure S6. Sanger sequencing validation for FiNCS in cell line MCF7 only detected from RNA-Seq data from CCLE and ENCODE but not in Weber et al. 2022.** Gene structures are shown in the reference tracks and fusion structures are shown in the fusion tracks. Sequences at the fusion breakpoints are shown above the Sanger sequencing chromatograms.


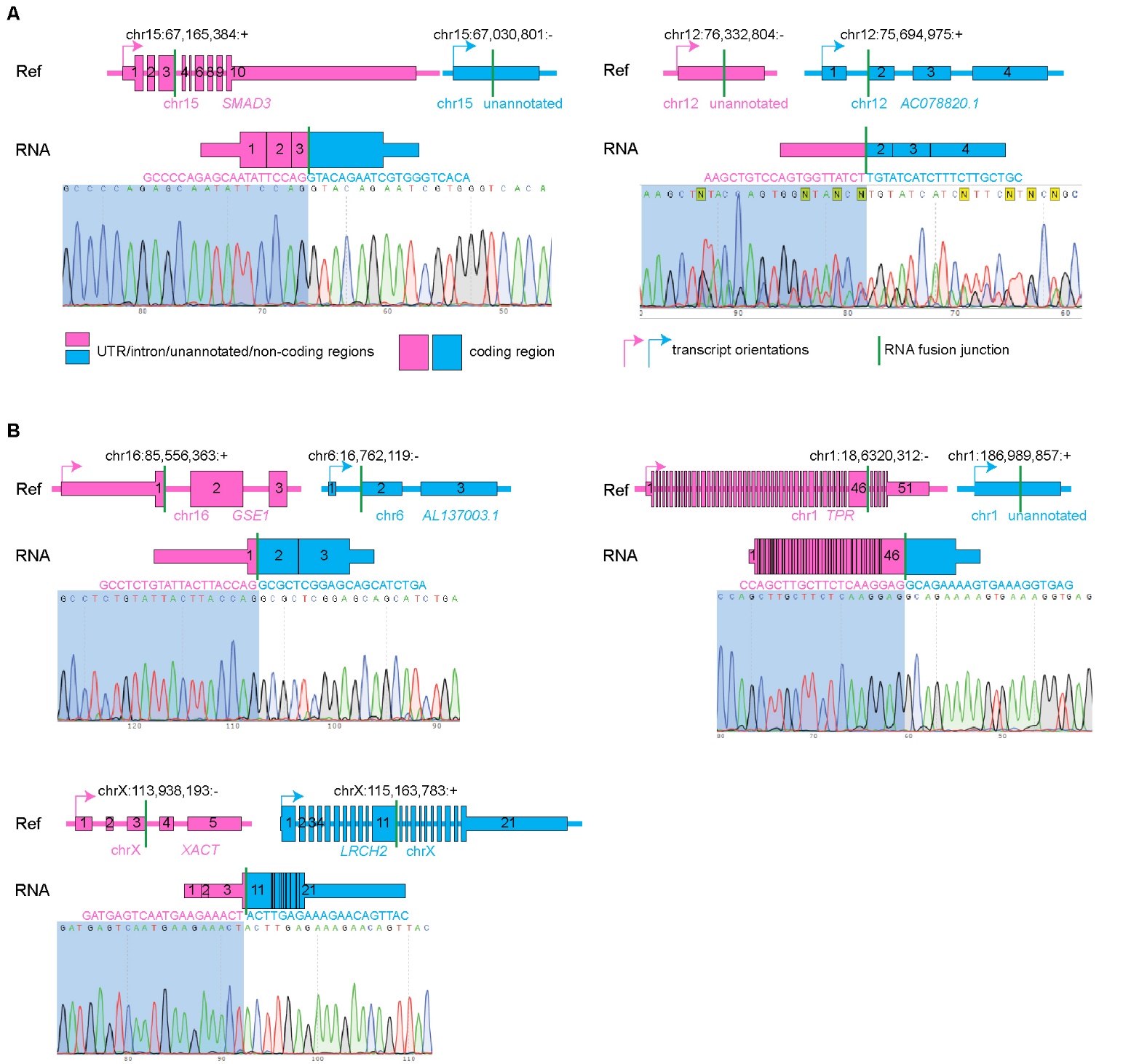


**Figure S7. Sanger sequencing validation for FiNCS in cell lines HCT116 and K562.** (**A**) FiNCS in HCT116. (**B**) FiNCS in K562. Gene structures are shown in the reference tracks and fusion structures are shown in the fusion tracks. Sequences at the fusion breakpoints are shown above the Sanger sequencing chromatograms.


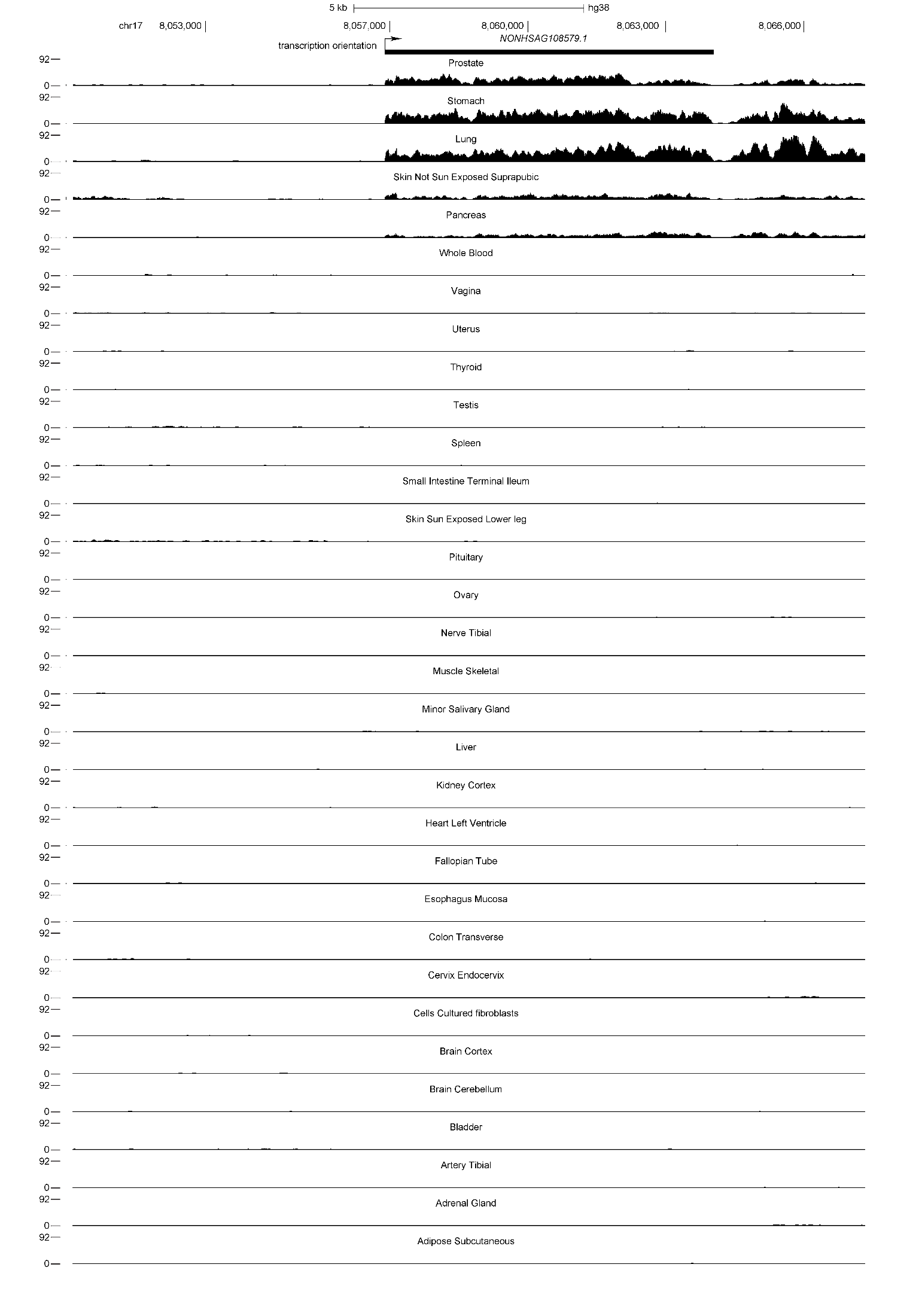


**Figure S8. Expression of *NONHSAG108579.1* in normal tissues.** The expression of *NONHSAG108579.1* and its flanking regions is shown in 32 normal tissues profile by GTEX.


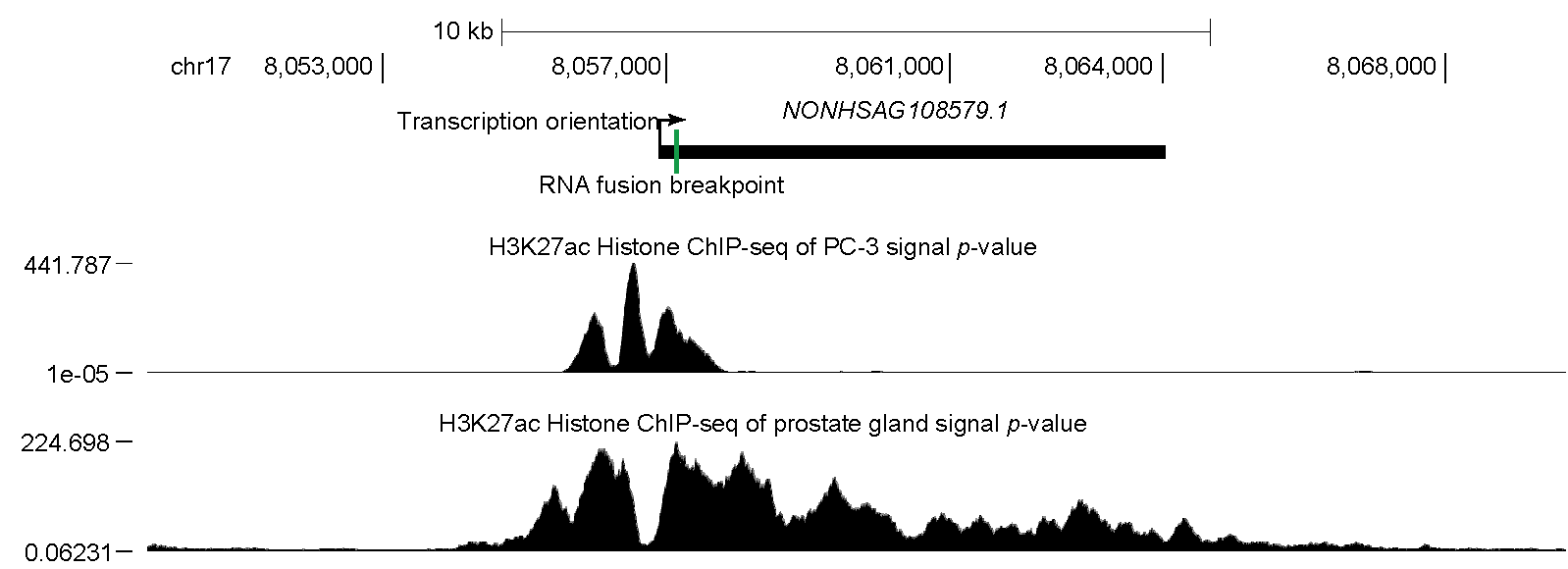


**Figure S9. H3K27ac signals at the *NONHSAG108579.1* locus for prostate cancer cell line PC-3 and normal prostate gland.**


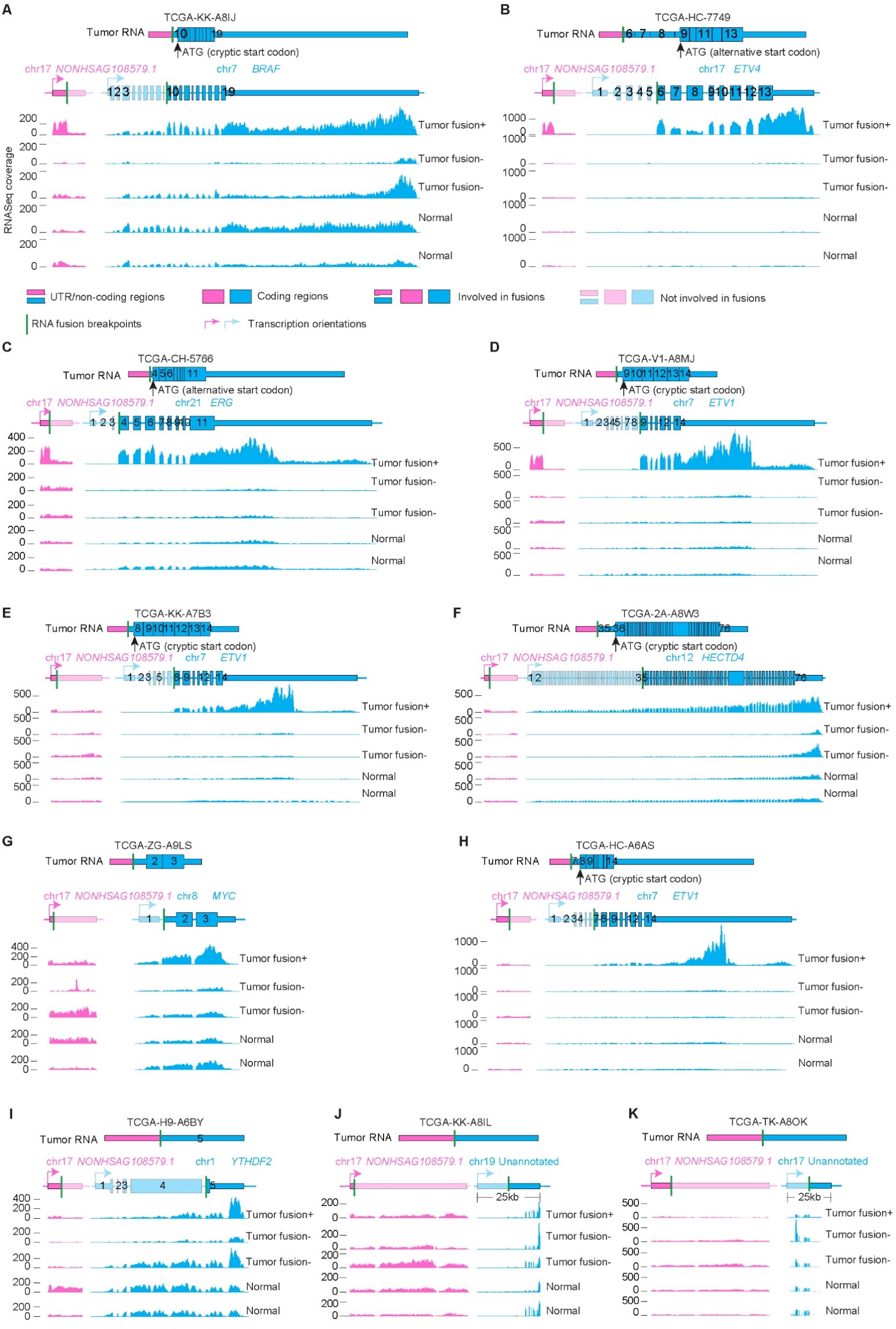


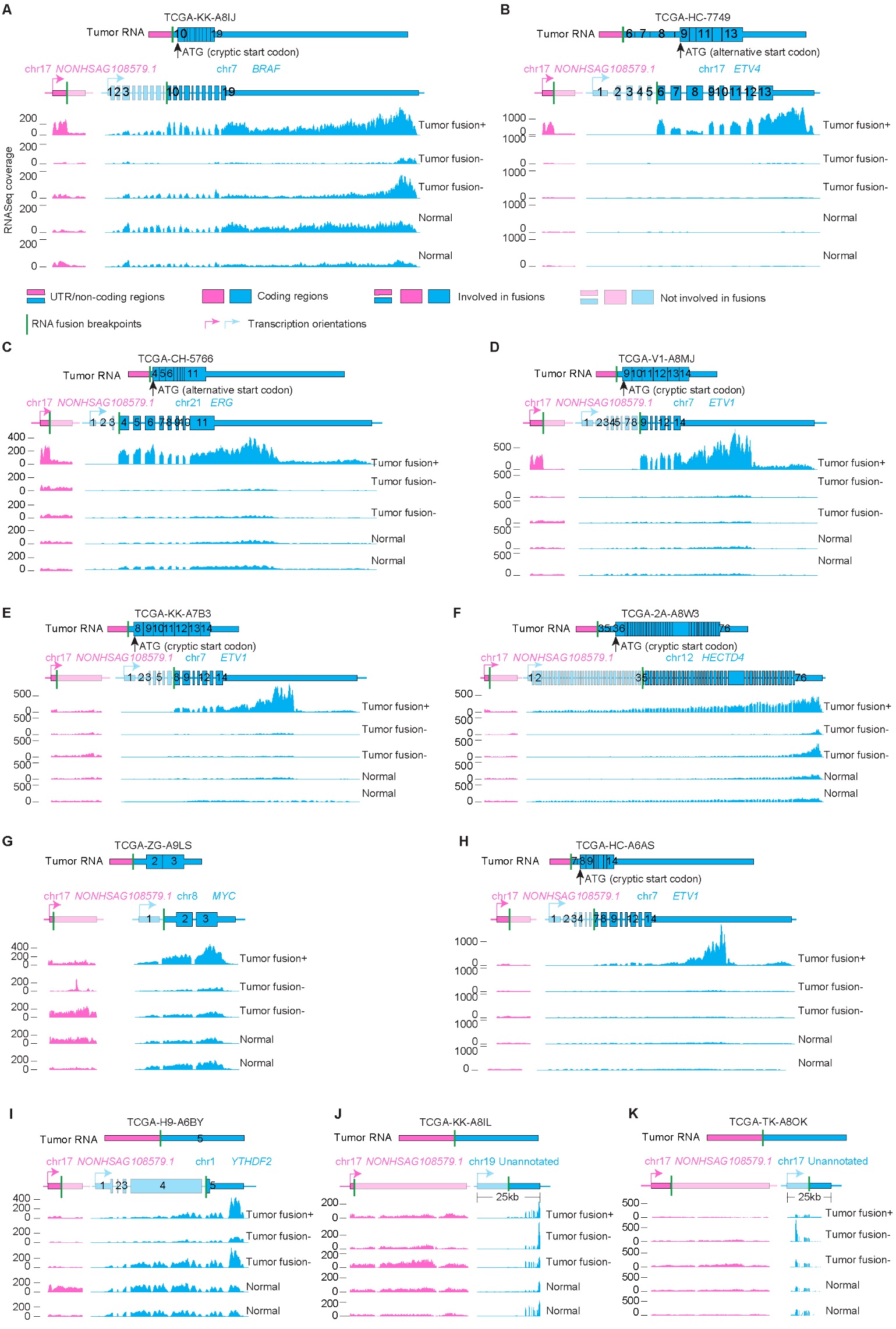


**Figure S10. Additional *NONHSAG108579.1* fusions in prostate cancers and their expression.** (**A**-**K**) Fusion structures are given on the top. Five tracks of RNA-Seq coverage are shown for five samples at the bottom and the reference gene structures are given above the five tracks. Exons and introns are re-scaled to better illustrate fusion structures. In A, the tumor samples without fusions (fusion-) are TCGA-HI-7169-01A-11R-2118-07 and TCGA-G9-6365-01A-11R-1789-07, and the normal samples are TCGA-EJ-7123-11A-01R-1965-07 and TCGA-EJ-7125-11A-01R-1965-07. In B, the fusion- samples are TCGA-HI-7169-01A-11R-2118-07 and TCGA-G9-6365-01A-11R-1789-07, and the normal samples are TCGA-EJ-7123-11A-01R-1965-07 and TCGA-EJ-7125-11A-01R-1965-07. In C, the fusion- samples are TCGA-FC-A5OB-01A-11R-A29R-07 and TCGA-V1-A9OL-01A-11R-A41O-07, and the normal samples are TCGA-EJ-A8FO-11A-11R-A36G-07 and TCGA-EJ-7331-11A-01R-2118-07. In D, the fusion- samples are TCGA-G9-6365-01A-11R-1789-07 and TCGA-V1-A9OL-01A-11R-A41O-07, and the normal samples are TCGA-EJ-A8FO-11A-11R-A36G-07 and TCGA-EJ-7331-11A-01R-2118-07. In E, the fusion- samples are TCGA-G9-6365-01A-11R-1789-07 and TCGA-V1-A9OL-01A-11R-A41O-07, and the normal samples are TCGA-EJ-A8FO-11A-11R-A36G-07 and TCGA-EJ-7331-11A-01R-2118-07. In F, the fusion- samples are TCGA-HI-7169-01A-11R-2118-07 and TCGA-G9-6365-01A-11R-1789-07, and the normal samples are TCGA-EJ-A8FO-11A-11R-A36G-07 and TCGA-EJ-7331-11A-01R-2118-07. In G, the fusion- samples are TCGA-HI-7169-01A-11R-2118-07 and TCGA-EJ-A7NJ-01A-22R-A352-07, and the normal samples are TCGA-EJ-7327-11A-01R-2118-07 and TCGA-HC-7742-11A-01R-2118-07. In H, the fusion- samples are TCGA-G9-6365-01A-11R-1789-07 and TCGA-V1-A9OL-01A-11R-A41O-07, and the normal samples are TCGA-EJ-A8FO-11A-11R-A36G-07 and TCGA-EJ-7331-11A-01R-2118-07. In I, the fusion- samples are TCGA-HI-7169-01A-11R-2118-07 and TCGA-G9-6365-01A-11R-1789-07, and the normal samples are TCGA-EJ-7327-11A-01R-2118-07 and TCGA-EJ-7331-11A-01R-2118-07. In J, the fusion- samples are TCGA-G9-6365-01A-11R-1789-07 and TCGA-V1-A9OL-01A-11R-A41O-07, and the normal samples are TCGA-EJ-A8FO-11A-11R-A36G-07 and TCGA-EJ-7331-11A-01R-2118-07. In K, the fusion- samples are TCGA-G9-6365-01A-11R-1789-07 and TCGA-V1-A9OL-01A-11R-A41O-07, and the normal samples are TCGA-EJ-A8FO-11A-11R-A36G-07 and TCGA-EJ-7331-11A-01R-2118-07.


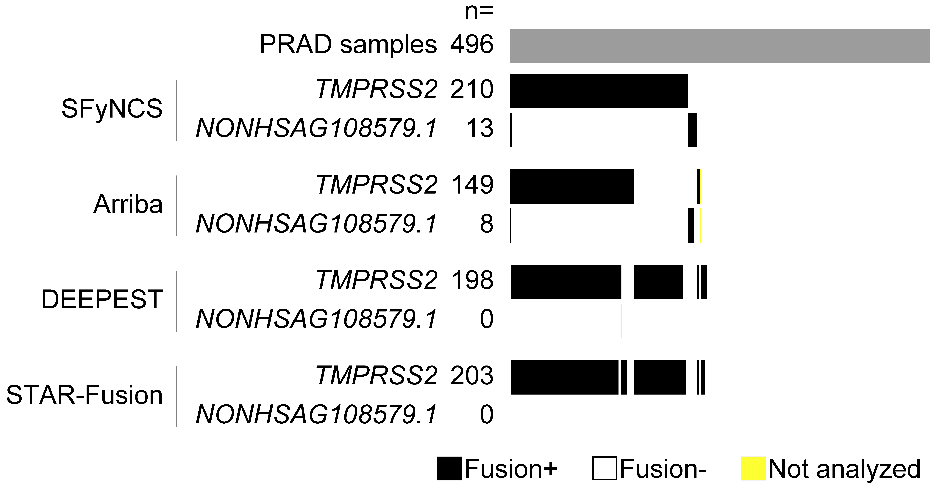


**Figure S11. Driver fusions detected by different tools in PRAD.** Numbers of fusions detected by individual tools are shown on the right of the gene names.


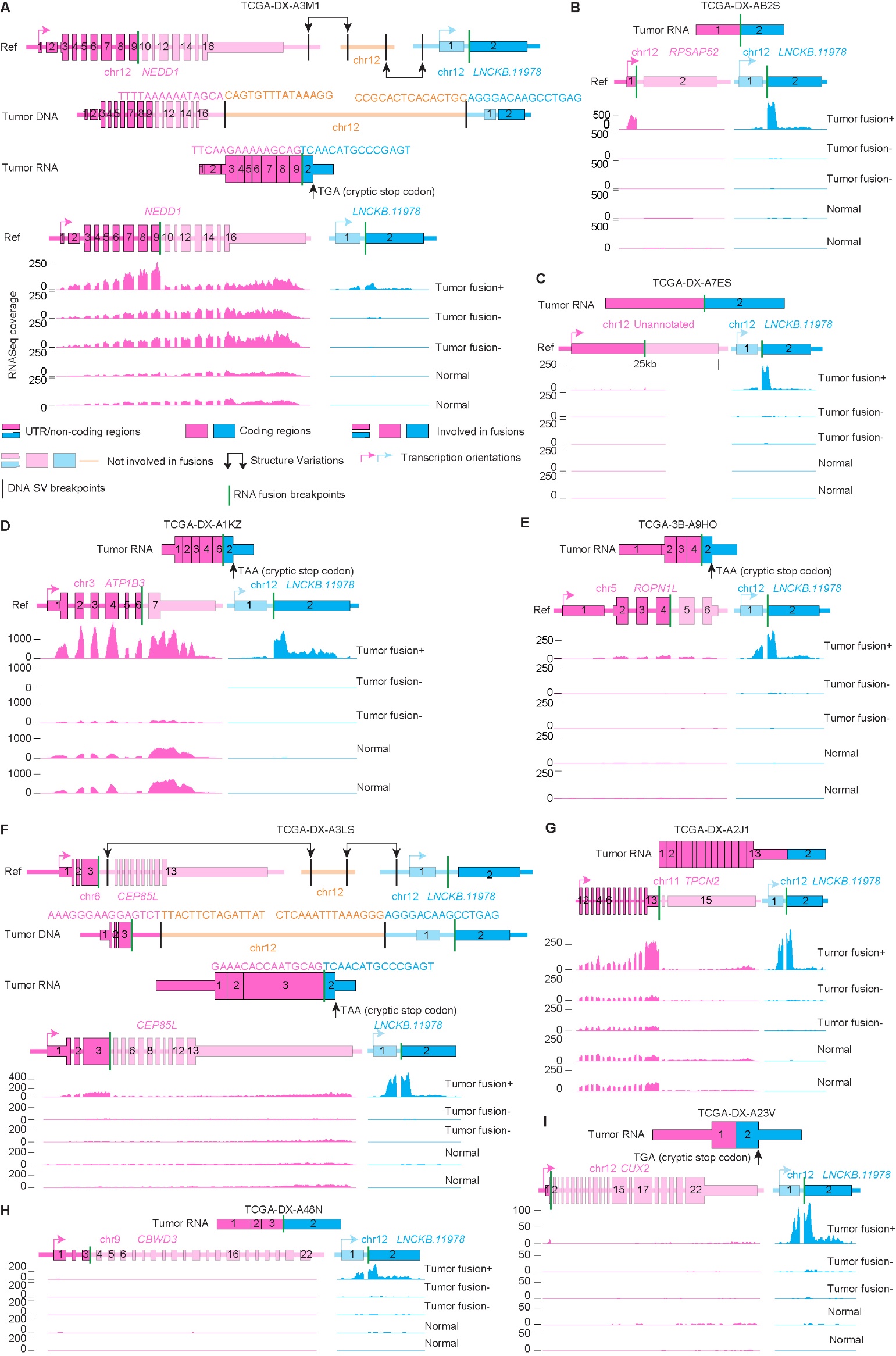


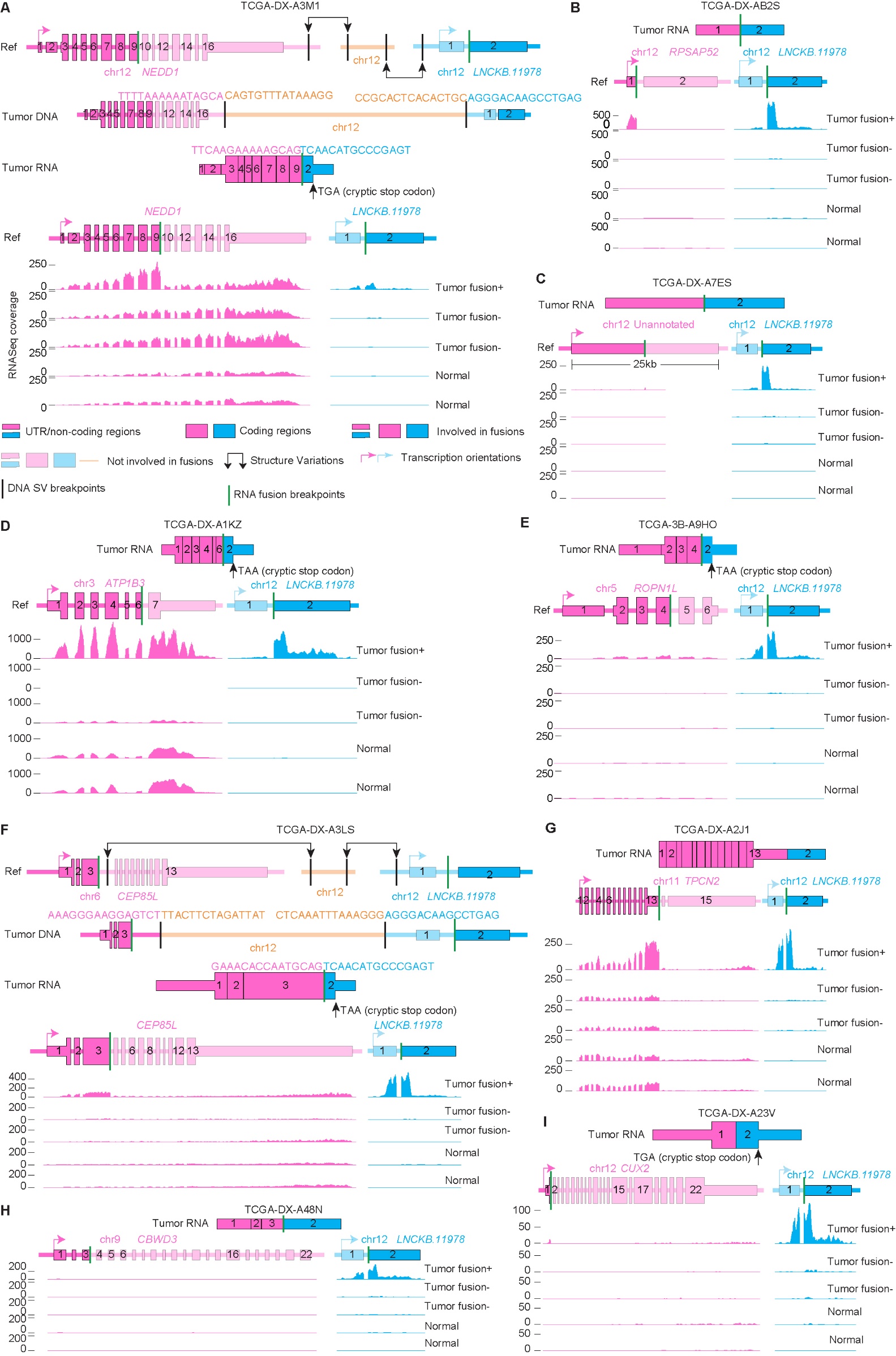


**Figure S12.** **Additional *LNCKB.11978*** **fusions in DDLPS and their expression. (A**-**I)**, Samples with WGS and RNA-Seq. Gene and fusion structures of reference genome, tumor DNA, and tumor RNA are shown as the top three tracks. In all cases, five tracks of RNA-Seq coverage are shown for five samples at the bottom and the reference gene structures are given above the five tracks. Exons and introns are re-scaled to better illustrate fusion structures. In A-I, the tumor samples without fusions (fusion-) are TCGA-IE-A4EI-01A-11R-A24X-07 and TCGA-IW-A3M4-01A-11R-A21T-07, and the normal samples are SRR1485722 and SRR1498838.


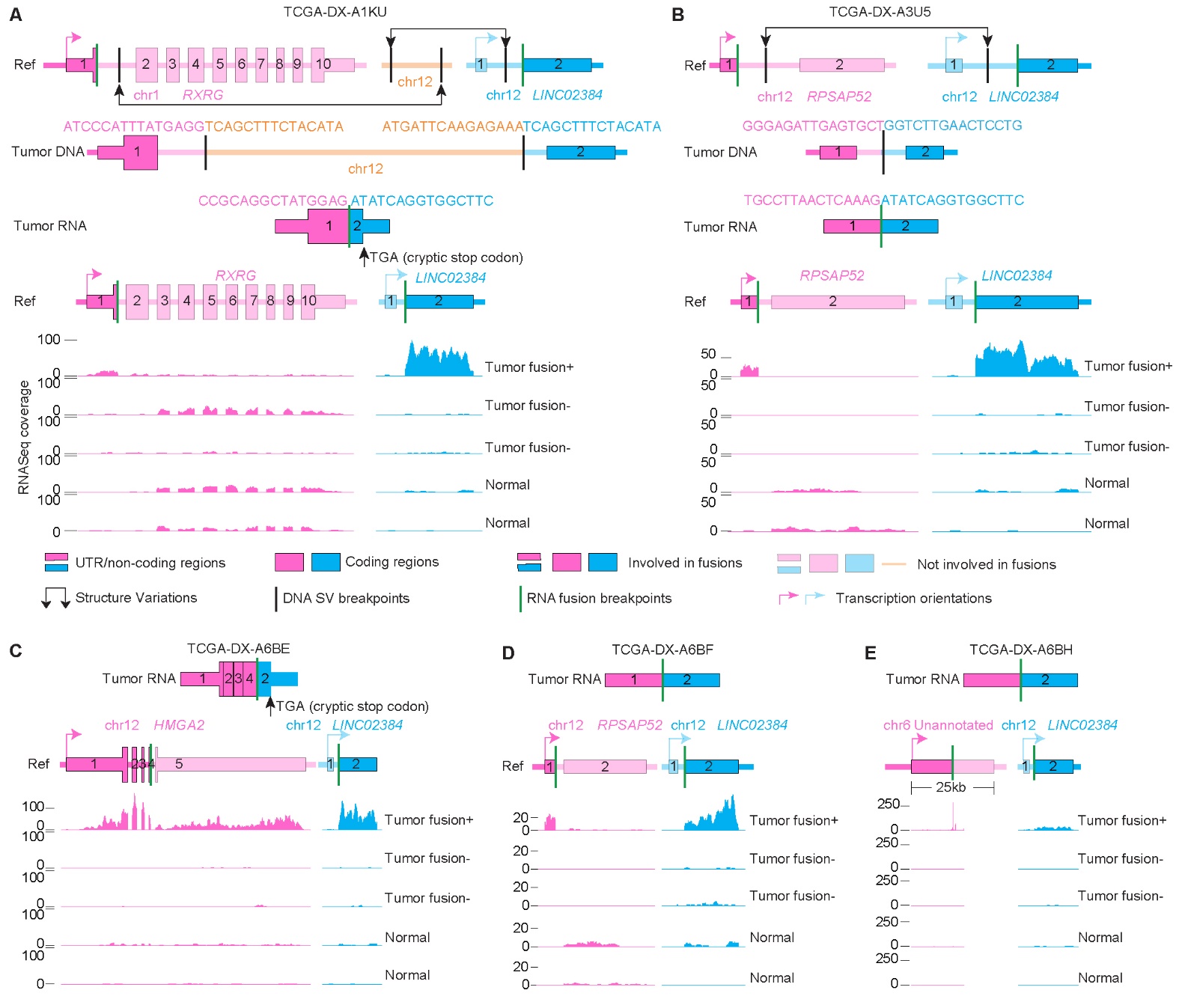


**Figure S13.** **Additional *LINC02384*** **fusions in DDLPS and their expression. (A-E)**, Samples with WGS and RNA-Seq. Gene and fusion structures of reference genome, tumor DNA, and tumor RNA are shown as the top three tracks. In all cases, five tracks of RNA-Seq coverage are shown for five samples at the bottom and the reference gene structures are given above the five tracks. Exons and introns are re-scaled to better illustrate fusion structures. In A-E, the tumor samples without fusions (fusion-) are TCGA-IE-A4EI-01A-11R-A24X-07 and TCGA-IW-A3M4-01A-11R-A21T-07, and the normal samples are SRR1485722 and SRR1498838.


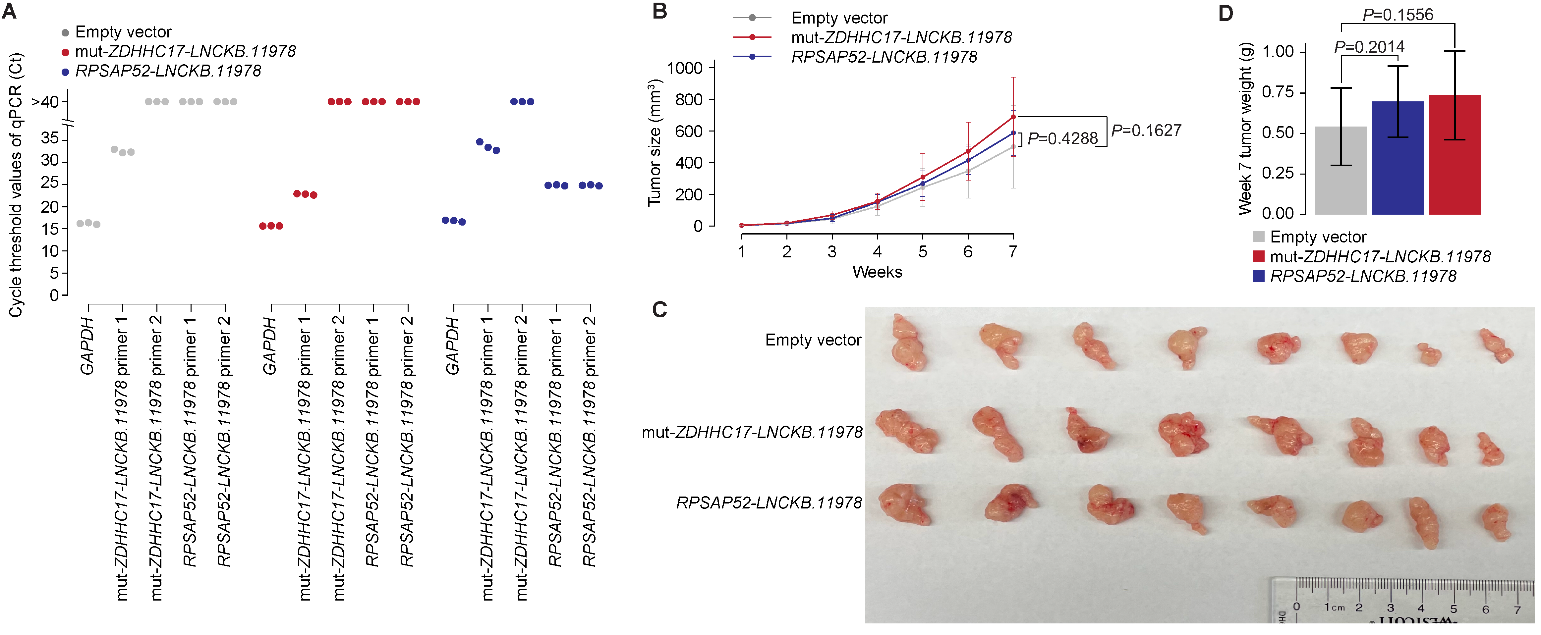


**Figure S14.** **Oncogenic functions of mut-*ZDHHC17*-*LNCKB.11978* and *RPSAP52-LNCKB.11978* fusion transcripts. (A)** Quantitative PCR showing the presence of mut-*ZDHHC17*-*LNCKB.11978* and *RPSAP52-LNCKB.11978* fusion transcripts in A549 cells. (**B**) Tumor growth curves after subcutaneous injection from week 1 to week 7. Error bars are standard deviations. *P* values are calculated by two-sided Student’s t-test. (**C**) Pictures of 8 tumors and tumor weights at week 7 after subcutaneous injection. (**D**) Tumor weights 7 weeks after injection. Error bars are standard deviations. *P* values are calculated by two-sided Student’s t-test.
