# Supplementary material for: SFyNCS detects oncogenic fusions involving non-coding sequences in cancer
Source: Nucleic Acids Res. 2023 Aug 28;51(18):e96. doi: 10.1093/nar/gkad705 (PMC10570049; doi:10.1093/nar/gkad705)
Supplement: gkad705_Supplemental_Files [file gkad705_supplemental_files.zip › SFyNCS.supplementary.table.legends.docx]

**Table S1. A subset of filtering parameters tested in benchmarking samples.** Each line represents one set of parameters. The line colored in pink is the parameters used in this manuscript. The remaining lines show filtering performances of altering one or a few parameters (colored in grey).

**Table S2. List of TCGA normal samples.** The table shows individual normal samples in the entire cohort and whether the samples belonged to the 140-sample normal panel.

**Table S3. List of fusions on which PCR and Sanger sequencing validation was performed.** Fusion breakpoints, primer sequences, cell lines, sources of fusions and whether the fusions were validated by PCR and Sanger sequencing are shown.

**Table S4. Fusion sequences synthesized for functional validation.**

**Table S5. The fusion statistics at tumor type level for all TCGA samples.** The table shows the numbers of tumor and normal samples, the numbers of tumor samples belonging to benchmarking samples, as well as the numbers of protein-coding fusion and FiNCS in each tumor type.

**Table S6. List of fusions in 338 TCGA benchmarking samples.** The fusions were identified by SFyNCS, Defuse (v0.8.1), FusionCatcher (v1.33), InFusion (v0.8.1-dev), and SQUID (v1.5) in 338 TCGA benchmarking samples. Note SQUID failed to analyze TCGA-DX-A2IZ-01A-11R-A21T-07.

**Table S7. List of fusions in MCF7 cell line.** The fusions were identified by SFyNCS in Weber et al. 2022, CCLE and ENCODE datasets.

**Table S8. List of fusions in HCT116 cell line.** The fusions were identified by SFyNCS in CCLE and ENCODE datasets.

**Table S9. List of fusions in K562 cell line.** The fusions were identified by SFyNCS in CCLE and ENCODE datasets.

**Table S10. List of fusions in all TCGA samples.** The fusions were identified by SFyNCS in all 9,565 TCGA samples.

**Table S11. The fusion statistics at sample level for all TCGA samples.** The table shows the numbers of fusions, the numbers of protein-coding fusions, the numbers of FiNCS in each of the 9,565 TCGA sample, as well as whether the samples belonged to the benchmarking samples.

**Table S12. List of recurrent fusion breakpoints.** The table shows the fusion breakpoints that occur in at least 3 samples in each tumor type. The breakpoint is annotated as protein-coding breakpoint if it belongs to protein-coding gene, otherwise, it would be non-coding breakpoint.

**Table S13. List of *NONHSAG108579.1* fusions in PRAD.** The table shows the samples containing the recurrent *NONHSAG108579.1* fusion breakpoint (chr17:8,057,148) in PRAD.

**Table S14. List of *lnckb.11978* and *LINC02384* fusions in SARC.** The table shows the samples containing the recurrent *lnckb.11978* (chr12:57890313) or *LINC02384* (chr12:68,333,664) fusion breakpoints in SARC.
